# Contribution to understanding the evolution of holometaboly: transformation of internal head structures during the metamorphosis in the green lacewing *Chrysopa pallens* (Neuroptera: Chrysopidae)

**DOI:** 10.1186/s12862-020-01643-2

**Published:** 2020-06-29

**Authors:** Chenjing Zhao, Yuchen Ang, Mengqing Wang, Caixia Gao, Kuiyan Zhang, Chufei Tang, Xingyue Liu, Min Li, Ding Yang, Rudolf Meier

**Affiliations:** 1 Department of Biology, Taiyuan Normal University, Jinzhong, 030619 China; 2 Department of Entomology, China Agricultural University, Beijing, China; 3 Department of Biological Sciences, National University of Singapore, Singapore, 117543 Singapore; 4 Institute of Plant Protection, Chinese Academy of Agricultural Sciences, Beijing, China; 5 Institute of Zoology, Chinese Academy of Sciences, Beijing, 100080 China; 6 Institute of Leisure Agriculture, Jiangsu Academy of Agricultural Sciences, Nanjing, 210014 China

**Keywords:** Chrysopidae, Neuroptera, 3D reconstruction, Transformation

## Abstract

**Background:**

Metamorphosis remains one of the most complicated and poorly understood processes in insects. This is particularly so for the very dynamic transformations that take place within the pupal sheath of holometabolous insects. Only few studies address these transformations especially with regard to cranial structures of those holometabolous species where the larval and adult forms have a similar diet. It thus remains unclear to what extent the internal structures undergo histolysis and rebuilding. Here, the development of the brain and skeleto-muscular system of the head of *Chrysopa pallens* (Rambur, 1838) is studied. This species is a predator of aphids in the larval and adult stage.

**Results:**

We used micro-computed-tomography (μ-CT) to study the transformations of the larval, prepupal and pupal head within the cocoon. We first assessed the morphological differences and similarities between the stages. We then determined the point in time when the compound eyes appear and describe the re-orientation of the head capsule which transforms the prognathous larva into a hypognathous adult. The internal head muscles are distinctly more slender in larvae than adults. In addition, the adults have a significantly larger brain which is likely needed for the processing of the signals obtained by the adults vastly expanded sensory organs that are presumably needed for dispersal and mating. Our study shows that the histolysis and modification of the inner muscles and skeletal elements take place within the prepupa. The central nervous system persists throughout metamorphosis but its morphology changes significantly.

**Conclusion:**

Our study reveals that not only the inner structures, but also the outer morphology continues to change after the final larval moult. The adult cuticle and internal structures form gradually within the cocoon. The histolysis and rebuilding begin with the skeletal elements and is followed by changes in the central nervous system before it concludes with modifications of the musculature. This order of events is likely ancestral for Holometabola because it is also known from Hymenoptera, Diptera, Mecoptera, and Coleoptera.

## Background

Metamorphosis is arguably one of the most interesting topics in insect evolution. It is interesting from a morphologically point of view because it often involves extreme modifications within the lifetime of the same individual, but metamorphosis is also important from an ecological point of view because larvae and adults of many insect species occupy very different ecological niches. All this diversity has evolved from a common ancestor, but the evolutionary trajectories are poorly understood and somewhat hidden behind the summary terms holometaboly, hemimetaboly, and ametaboly. The diversity of developmental mechanisms is particularly poorly understood for holometabolous insects which have a pupal stage. This stage is used for the re-organization of the larval body into an adult with often dramatically different morphology. The transformation is so remarkable that a number of model species have been studied in great detail (e.g., *Drosophila melanogaster*, *Tenebrio molitor, Apis mellifera*). However, Holometabola also include taxa where the morphological and ecological transformations are much less pronounced. It is for these taxa that comparatively little data are available. This lack of information interferes with understanding the origin of the kind of extreme metamorphosis observed in the model taxa. One example for a holometabolous species with similar feeding ecology as larva and adult is the green lacewing *Chrysopa pallens* which is a predator of aphids throughout its life. We here study the development of this species.

Haug [1] recently discussed criteria that could be used to characterize the term “larva”, but the same classification is also useful for identifying major questions in the evolution of metamorphosis. When classified based on morphology, Haug distinguishes morpho-larvae sensu *lato* from morpho-larvae sensu stricto depending on whether the structures that distinguish the larvae from the adults are lost during development. According to Haug’s classification, only those morpho-larvae that lose the larval features are considered morpho-larva s.s.. When compared to the ancestor, the larval features can either be derived (“apo-larva”) or all the novelty is found in the adults which then renders the immature stage a “plesio-larva” sensu Haug (although it is likely that few larvae are entirely “apo” or “plesio”). Haug’s characterization of the term “larva” also considers ecology. Eco-larvae s.l. and eco-larvae s.s. occupy ecological niches that are different from those of the adults, but only in the case of eco-larvae s.s., the larvae are also responsible for dispersal.

Holometabola (= Endopterygota) comprises with approximately 800,000 described species about two thirds of the described animal species [2]. Haug’s criteria can be used to explore the developmental space that is occupied by holometabolan insects. At one end of the spectrum, one may expect species with immature stages that lack discrete features that distinguish them from the corresponding adults (i.e., species without larvae), but such species are apparently not known from insects so that all holometabolan species have at least morpho-larvae sensu *lato* [1]. Similarly, rare or missing in Holometabola are eco-larvae sensu *strictu* because the dispersal stage of endopterygote insects is the adults. Most holometabolan insect species thus occupy fairly extreme points in the developmental space. They have morpho-larvae with usually numerous apomorphic features and the larvae and adults of most species occupy different ecological niches. The degree of differentiation differs greatly between and within clades. A species like the neuropteran, *Chrysopa pallens*, has a comparatively moderate metamorphosis compared to what is observed in extreme cases like *Drosophila*. Holometabolous insects may all have a pupa but the extent to which the larval body has to be re-modelled varies widely.

The pupal stage of Holometabola enables some of the most drastic metamorphosis in Metazoa [3]. Some authors consider the pupal stage a specialized larva [4], but alternatively the pupa has also been thought to correspond to the nymphal stage, in which case the larva would then be a so-called pro-nymph [5]. One common belief is that the metamorphosis in holometabolous insects occurs in two more or less discrete steps: a) metamorphosis of the external parts which is supposed to be completed with the shedding of the last larval cuticle which is thought to remain largely unchanged during the pupal stage, and b) the internal transformation occurring inside the pupa [6–8]. Saltin et al. [9] tested this assumption and found that the two-step model is an oversimplification. They show for mecopteran *Panorpa* species that the adult cuticle inside the pupa develops gradually during the late pupal stage.

Overall, the evolution of holometabolan metamorphosis is fairly poorly understood because too few species have been studied in sufficient detail. The most detailed descriptions tend to be available for species with extreme metamorphoses. Hymenoptera are the sister group of all remaining Holometabola [10]. For this order, good information is available for the honey bee, *Apis mellifera*. Oertel [11] provided a detailed account of the transformations of the skeleton, digestive, and muscular systems, but unfortunately provides less detail on cephalic musculature. A second major clade of Holometabola consists of Strepsiptera, and Coleoptera. The metamorphosis of the mealworm *Tenebrio molitor* is well studied for the nerve system [12, 13], but Ge et al. [14] also reconstructed the skeleto-muscular system of head of the final instar larvae, day 4 pupae, and adults of chrysomeline species. In addition, Polilov & Beutel [15, 16] compared changes in the hooded beetle *Sericoderus lateralis* (Coleoptera: Corylophidae) and *Mikado* sp. (Coleoptera: Ptiliidae). The latter studies covered larvae, pupae and adults, but the focus of the studies was the effects of miniaturization on morphology and the use of the characters for a phylogenetic reconstruction. Within the Antliophora (Diptera, Mecoptera, Siphonaptera), the best description of development is available for *Drosophila melanogaster* Meigen, 1830 [17] but the genesis of the adult abdominal muscles in blowflies is also well characterized [18]. For Mecoptera, Saltin et al. [9] studied the morphogenetic processes that lead to adult cuticle in the mecopteran species *Panorpa vulgaris* Imhoff & Labram, 1838 and *Panorpa communis* Linnaeus, 1758. Within the Amphiesmenoptera (Lepidoptera, Trichoptera), Lowe et al. [19] reconstructed the complete development of the tracheal system and midgut of the painted lady (*Vanessa cardui*) (Lepidoptera: Nymphalidae). However, unfortunately several order-level clades of Holometabola still lack detailed information on metamorphosis. This includes Neuroptera, Megaloptera, and Raphidioptera.

Here we present new data on the head development of a neuropteran, a green lacewing species which has a special prepupal stage at the late final instar larva phase within the cocoon before the pupa is formed. We reveal how the skeleto-muscular system and central nervous system of the adult head is formed within the cocoon. Our study provides a model for the metamorphosis for Neuroptera. We compare the metamorphosis of a lacewing species with what is known for other endopterygote species with a focus on those organs that are most extensively rebuilt. In order to study the soft tissues within the pupa, we used micro-computed-tomography (μ-CT), which has been shown to be a valuable tool for the study of insect anatomy [20–22]. We here focus on the skeleto-muscular and central nervous system of the head. Other character systems such as digestive system and tracheal system will be described later.

Green lacewings are one of the most commonly encountered neuropterans. Lacewing species differ considerably with regard to morphology and many species have highly specialized life histories, particularly as larvae. They are found in all major biogeographic regions of the world [23, 24]. *Chrysopa pallens* (Rambur, 1838) belongs to Chrysopidae within Neuroptera. The larvae of this species are typical for Neuroptera in that they are highly derived predators with unique mouth parts that form venom-injecting stylets and sucking mouthparts. These mouthparts have been considered a key innovation that is partially responsible for the evolutionary success of neuropterans [25–27]. Note that some neuropterans also play an important role as biological control agents of insect pests such as aphids in agriculture [28–31]. In particular, several species in the genera *Chrysopa* and *Chrysoperla* [(e.g., *Chrysopa pallens*, *Chrysoperla carnea* (Stephens)] are mass-reared and sold by commercial insectaries. The reasons why we chose *Chrysopa pallens* for our study was the lack of developmental information on Neuroptera, the fact that adults and larvae have the same diet, and the availability of fresh material from cultures.

The morphological studies on immature stage of green lacewing cover external structures of larvae [32–34] while information on internal structures such as musculature are lacking. For example, there is no description of the anatomy of pupae and transformation of the internal structures during metamorphosis for green lacewings. However, Miller [35] provided morphological information on the adult cephalic and thoracic musculature of *Chrysopa plorabunda* based on traditional anatomical methods, even if some muscles were apparently overlooked such as tentoriomandibularis muscles, and some muscles were subsumed under one term whereas they were treated as separate units by Wipfler et al. [36]. In the present study, we document the muscular system and the central nervous system of head capsule from larvae to adults using micro-CT.

## Results

Most neuropterans have only three larval stages; i.e., the third is the last one. After cocooning, we labelled the specimens by day of collection: Day 1 to Day 12 until the adults emerge. The cranial musculoskeletal system and the cephalic nervous system of the third instar larvae (including the prepupal phase, from Day 1 to Day 4), the pupae (from Day 5 to Day 12), and the adults of *Chrysopa pallens* were reconstructed and described. In the skeletal system of the larval head, we focused more on the outer cuticle. For the pupal head, we focus on the inner cuticle.

### Skeleto-muscular system

The external structures of head are described. For the prepupae (i.e. specimens from Day 1 to Day 4), the skeletal system is almost identical to what is found in the 3rd instar larvae, thus only the latter is described in detail. At Day 5, the larval cuticle cracked, and the pupa develops gradually during the following 7 days. We here only describe the well-developed pupae as found on the 11th day. The transformation of muscles inside the cocoon are also described. The description of head muscles in the 3rd larva, Day 11 pupa, and adult (♂) is also presented in Tables 1, 2 and 3, respectively.

**Table 1:** Cephalic musculature of the larvae of *Chrysopa pallens* (Rambur, 1838)

Muscle Abb./No.	Origin	Insertion	Presumed function
Labrum/2			
0 lb1	Mesally on the frons	Mesally on the basal wall of the labrum	levator of labrum
0 lb2	Laterally on the frons	tormae	levator of labrum
Antenna/4			
0an1	dorsal tentorial arms	Anterior antennal base (ventral)	depressor and flexor of antenna
0an2	dorsal tentorial arms	Posterior antennal base (dorsal)	levator of antenna
0an3	dorsal tentorial arms	Lateral antennal basal margin	
0an4	dorsal tentorial arms	Mesal antennal basal margin	
Mandible/3			
0md1	Posterior, lateral, and dorsal parts of the head capsule	Tendon that inserts at the median edge of the mandible	adductor of mandible
0md3	Lateral, ventral, and dorsal parts of the head capsule	Tendon that inserts at the lateral edge of the mandible	abductor of mandible
0md8	anterior tentorial arms	Mediodorsal wall of the mandibular cavity	adductor of mandible
Maxilla/6			
0mx2	Ventrolateral, anterolateral parts of the head capsule	Basal part of the maxilla stylet	adductor of maxilla stylet
0mx3	Proximal part of anterior tentorial arms	Cardo	protractor of maxilla
0mx4	Proximal part of the anterior tentorial arms	Anterior part of stipes	protractor of maxilla
0mx5	Anterior tentorial arms, anterior to the 0mx4	posterior part of stipes	protractor of maxilla
0mx6	stipes	Basal edge of the maxilla stylet	adductor of lacinia
imm	Basal part (dorsal) of the maxilla stylet	Basal part (ventral) of the maxilla stylet	
Labium/3			
0la5	Posterior tentorial arms	Posterolateral part of the prementum	adductor of praementum
0la8	Posterior region of the mentum	Posterior edge of the prementum	retractor of praementum
0la14	Anterior edge of the prementum	Basal part of the labial palp	levator of labial palp
Epipharynx/2			
0ci1	clypeus	Roof of the cibarium	dilatator ofcibarium
0bu1	Postclypeus	Roof of the bucca	dilator of buccal cavity
Hypopharynx/1			
0hy3	Posterolateral part of the head capsule	Anterolateral part of hypopharynx	levator of the hypopharynx
Pharynx/10			
0bu2	Middle region of the frons	Dorsal buccal wall	dilator of pharynx
0bu3	Frons, posterior to the 0bu2	Dorsal buccal wall	dilator of pharynx
0bu4	anterior tentorial arms	Lateral wall of the bucca	dilator of pharynx
0bu5	Tentorial bridge	Ventral wall of the bucca	dilator of pharynx
0bu6	Tentorial bridge	Ventral wall of the bucca	dilator of pharynx
prhy	Lateral region of the prementum	Anterior region of the ventral bucca	dilator of pharynx
0 ph 1	Posterior region of vertex	Dorsal wall the pharynx	
0 ph 2	Posterior tentorial arms	Ventral and lateral region of the pharynx	dilator of pharynx
0st1	Ring muscle layer that covers the entire pharynx		constrictor of the pharynx
0st2	Longitudinal muscle layer directly above musculus annularis stomodaei		constrictor of the pharynx

**Table 2:** Cephalic musculature of the pupae of *Chrysopa pallens* (Rambur, 1838)

Muscle Abb.	Origin	Insertion
Labrum/3		
0 lb1	Lateral on the frons, below the antennal base	Mesally on the outer basal wall of the labrum
0 lb2	Laterally on the frons	Tormae
0 lb4	Dorsal labral wall	Ventral labral wall
Antenna/4		
0an1	Anterior tentorial arms	Anterior basal margin of the scape
0an2	Anterior tentorial arms	Posterior basal margin of the scape
0an6	Dorsal wall of the scape	Lateral wall of the pedicel
0an7	Mesal wall of the scape	Mesal edge of the pedicel
Mandible/4		
0md1	The part between occipital and compound eyes of the head capsule	Tendon that inserts at the median edge of the mandible
0md3	Posterior part of head capsule, lateral region of the 0md1	Tendon that inserts at the lateral edge of the mandible
0md4	Lateral part of the hypopharynx	Inner side of the median edge of the mandible
0md8	Anterior tentorial arms	Mediodorsal wall of the mandibular cavity
Maxilla/11		
0mx1	Posterior part of the gena, close to the 0md1	Basal region of the cardo
0mx2	Post part of the gena	Basal region of the lacinia
0mx3	Ventral side of the anterior tentorial arms	cardo
0mx4	Ventral side of the anterior tentorial arms	Anterior edge of the stipes
0mx5	Ventral side of the anterior tentorial arms	Anterior edge of the stipes, close to the 0mx4
0mx6	Stipital base	Basal edge of the lacinia
0mx8	Inner wall of the stipes	Distal basal edge of the maxillary cardo
0mx12	Basal edge of palpomere 1	Basal edge of palpomere 2
0mx13	Basal edge of palpomere1	Basal edge of palpomere 3
0mx14	Basal edge of palpomere 3	Basal edge of palpomere 4
0mx15	Basal edge of palpomere 4	Basal edge of palpomere 5
Labium/3		
0la14	Basal edge of the prementum	Basal edge of the labial palpus
0la16	Inner wall of the palpomere 1	Basal edge of the palpomere 2
0la17	Inner wall of the palpomere 2	Basal edge of the palpomere 3
Epipharynx/2		
0ci1	Mesally on the clypeus	Posterior region of the epipharynx, covered by 0bu1
0bu1	Anterior region of frons	Roof of the bucca
Hypopharynx/3		
0hy1	frons	Oral arms of the suspensorial sclerites
0hy2	Anterior tentorial arm	Oral arms of the suspensorial sclerites
0hy12	Anterior part of the hypopharynx	Inner wall of the ligula
Pharynx/5		
0bu2	Frons, below the antennal base	Dorsal wall of the pharynx
0bu3	Posterior part of frons, lateral region of the antennal base	Dodrsal wall of the pharynx, posterior to 0bu2
0bu5	Anterior tentorial arms, anterior to tentorial bridge	Ventral wall of the pharynx
0bu6	Anterior tentorial arms, lateral to 0bu5	Ventral wall of the pharynx
0 ph 2	Posterior region of the tentorial bridge	Lateral wall of the pharynx

**Table 3:** Cephalic musculature of the adults of *Chrysopa pallens* (Rambur, 1838)

Muscle Abb.	Origin	Insertion	Presumed function
Labrum/3			
0 lb1	Frons	Mesally on the outer basal wall of the labrum	Levator of labrum
0 lb2	frons	tormae	Levator of labrum
0 lb4	Dorsal part of the labrum	Ventral part of the labrum	Compressor of labrum
Antenna/7			
0an1	Anterior tentorial arms	Anterior basal edge of the scape	Depressor and flexor of antenna
0an2	Anterior tentorial arms, posterior to 0an1	Posterior basal edge of the scape	Levator of antenna
0an4	Dorsal tentorial arms	Mesal basal edge of the scape	Depressor and rotator of antenna
0an6	Laterally, mesally of the dorsal wall of the scape	Lateral basal edge of the pedicel	Extensor of flagellum
0an7	Inner wall of scape	Basal edge of the pedicel	Flexor of flagellum
0an9	Ventral wall of the scape	Posteriro basal region of the pedicel	Depressor of the antenna
0an10	Dorsal wall of the scape	Anterior basal region of the pedicel	Elevator of the antenna
Mandible/5			
0md1	Posterior part between occipital and compound eye of the head capsule	Tendon that inserts at the median edge of the mandible	Adductor of mandible
0md3	Gena and postgena	Tendon that inserts at the lateral edge of the mandible	Abductor of mandible
0md4	Lateral wall of the hypopharynx	Inner median wall of the mandible	Protractor of anatomical mouth opening
0md6	Ventral side of the anterior tentorial arms	ventral basal margin of the mandible	Adductor of mandible
0md8	Anterial tentorial arms	Mediodorsal wall of the mandibular cavity	
Maxilla/13			
0mx1	Posterior part of the head capsule	Basal cardinal process	Promoter of maxilla
0mx2	Posterior part of gena	Basal part of the lacinia	Adductor of lacinia
0mx3	Ventral side of the anterior tentorial arms	Carostipital sulcus	Adductor of cardo and protractor of maxilla
0mx4	Ventral side of the anterior tentorial arms, under 0mx3	Anterior edge of the stipes	Adductor of maxilla
0mx5	Lateral part of tentorial bridge	Basally on the stipes, close to 0mx4	Adductor of stipes and protractor of maxilla
0mx6	Lateral wall of stipes, close to carostipital sulcus	Basal edge of the lacinia	Adductor of lacinia
0mx7	Mesal wall of stipes	Basal edge of the galea	Abductor of galea
0mx8	Lateral wall of the stipes, basal to the maxillary palpus	Basal edge of the first palpomere	Abductor of maxillary palp
0mx10	Stipital ridge	Distal edge of the palpomere 1	Adductor of maxillary palp
0mx12	Basal edge of palpomere 1	Basal edge of palpomere 2	Adductor of maxillary palpomere ii
0mx13	Basal edge of palpomere 1	Basal edge of palpomere 3	Abductor of maxillary palpomere iii
0mx14	Basal edge of palpomere 3	Basal edge of palpomere 4	Adductor of maxillary palpomere iv
0mx15	Basal edge of palpomere 4	Basal edge of palpomere 5	Adductor of maxillary palpomere v
Labium/6			
0la5	Posterior tentorial arms	Laterobasal edge of prementum	Adductor of praementum
0la8	Posterior part of submentum	Posterior edge of mentum	Retractor of praementum
0la13	Distally on the prementum	Distal edge of the labial palpus	Adductor of labial palpomere i
0la14	Basal edge of the prementum	Basal edge of the labial palpus	Levator of labial palp
0la16	Basal edge of palpomere 1	Basal edge of palpomere 2	Flexor of labial palpomere ii
0la17	Basal edge of palpomere 2	Basal edge of palpomere 3	Flexor of labial palpomere iii
Epipharynx/2			
0ci1	Mesally on the clypeus	Posterior region of epipharynx	Dilatator ofcibarium
0bu1	Anterior region of frons	Roof of the bucca	Dilator of buccal cavity
Hypopharynx/5			
0hy1	frons	Oral arms of the suspensorial sclerites	Levator and dilator of anatomical mouth
0hy2	Anterior tentorial arm	Oral arms of the suspensorial sclerites	Dilator of anatomical mouth
0hy8	Basal part of prementum	Lateral wall of salivarium	Dilator of salivarium
0hy9	Oral arm of suspensorial sclerite	Oral arm of the suspensorial sclerites on the other side	Connecting the anterior oral arms
0hy12	Anterior region of hypopharynx	Dorsolateral wall of salivarium	Dilator of salivarium
Pharynx/9			
0bu2	Frons, below the antennal base	Dorsal wall of pharynx	Dilator of pharynx
0bu3	Posterior region of frons, lateral to the antennal base	Dorsolateral wall of pharynx	Dilator of pharynx
0bu4	Anterior tentorial arms	Lateral wall of bucca	Dilator of pharynx
0bu5	Tentorial bridge	Ventral wall of pharynx	Dilator of pharynx
0bu6	Tentorial bridge	Ventral wall of pharynx	Dilator of pharynx
0 ph 1	Vertex	Dorsal wall of the pharynx	Dilator of pharynx
0 ph 2	Posterior tentorial arms	Lateral wall of pharynx	Dilator of pharynx
0st1	Ring muscle layer that covers the entire pharynx		Constrictor of the pharynx
0st2	Longitudinal muscle layer directly above musculus annularis stomodaei		Constrictor of the pharynx

#### General appearance

##### Third instar larvae (Fig. 1a)

Body of living third instar larvae fusiform and humped. Length ~ 7.00 mm and height ~ 1.30 mm. Cuticle light brown with dark brown markings dorsolaterally. Spinules and long microsetae present dorsally. All setae smooth, dark brown to light brown. Head dorsoventrally flattened 0.70 mm in length and 1.00 mm in width, with strongly prognathous prominent sucking stylets. Thorax unsclerotized with rows of short, acute setae. Legs slender and well developed, inserted on semi-membranous ventrolateral articulatory areas posteriorly. Lateral tubercles broadly short cylindrical dorsolaterally and tapering distally with elongated setae. Long setae all tapering and hooking at tips. Tubercles and long setae carry the debris for camouflage.

**Fig. 1:**
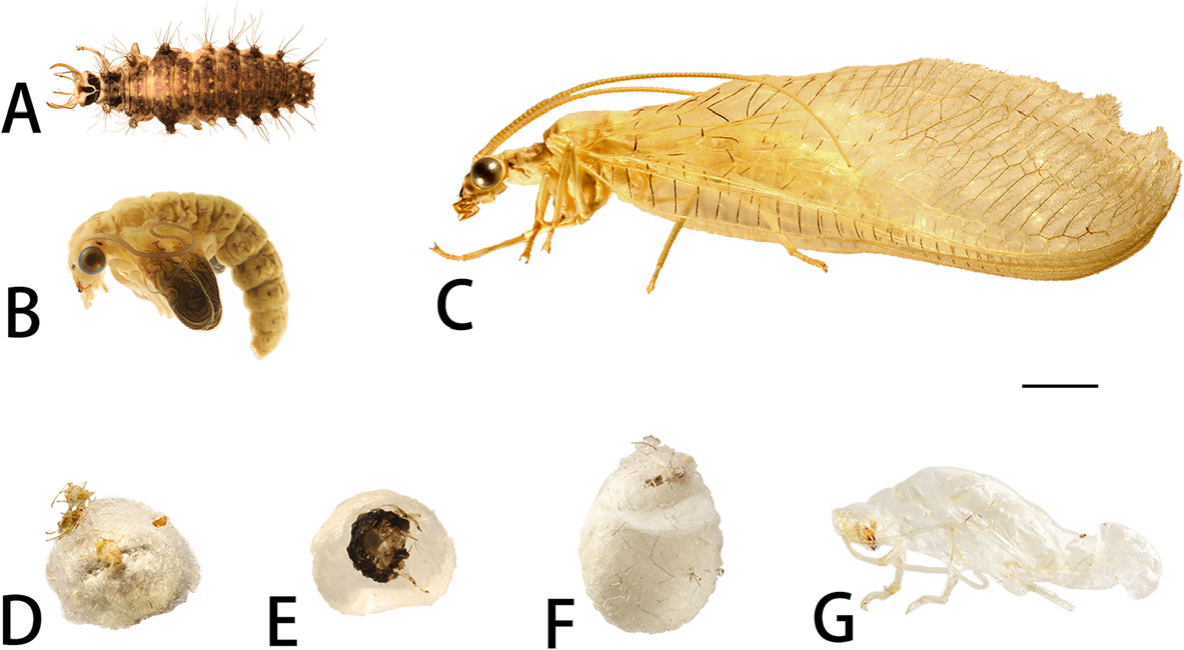
*Chrysopa pallens*, photographs: **a** larvae, dorsal view; **b** pupae (Day11, pupal sheath with pharate adult inside), lateral view; **c** adults (♂), lateral view; **d** cocoon of pupal stage; **e** cocoon of Day 6–11, inside view; **f** cocoon of Day 12; **g** cuticle of Day 12 pupa. Scale bar: 2 mm

##### Prepupae (Fig. 2). From day 1 to day 4

Prepupae immobile adecticous exarate type. Cuticle light brown-yellow. Body C-shape with 5.0 mm in length and 3.0 mm in width. Head bends inward, morphologically almost same to larvae. Segments of thorax and abdomen similar in shape. Lateral tubercles smaller and long setae disappeared. Cocoon 4.0 mm in length and 3.0 mm in width, with dead aphids covering the cocoon (Fig. 1d).

**Fig. 2:**
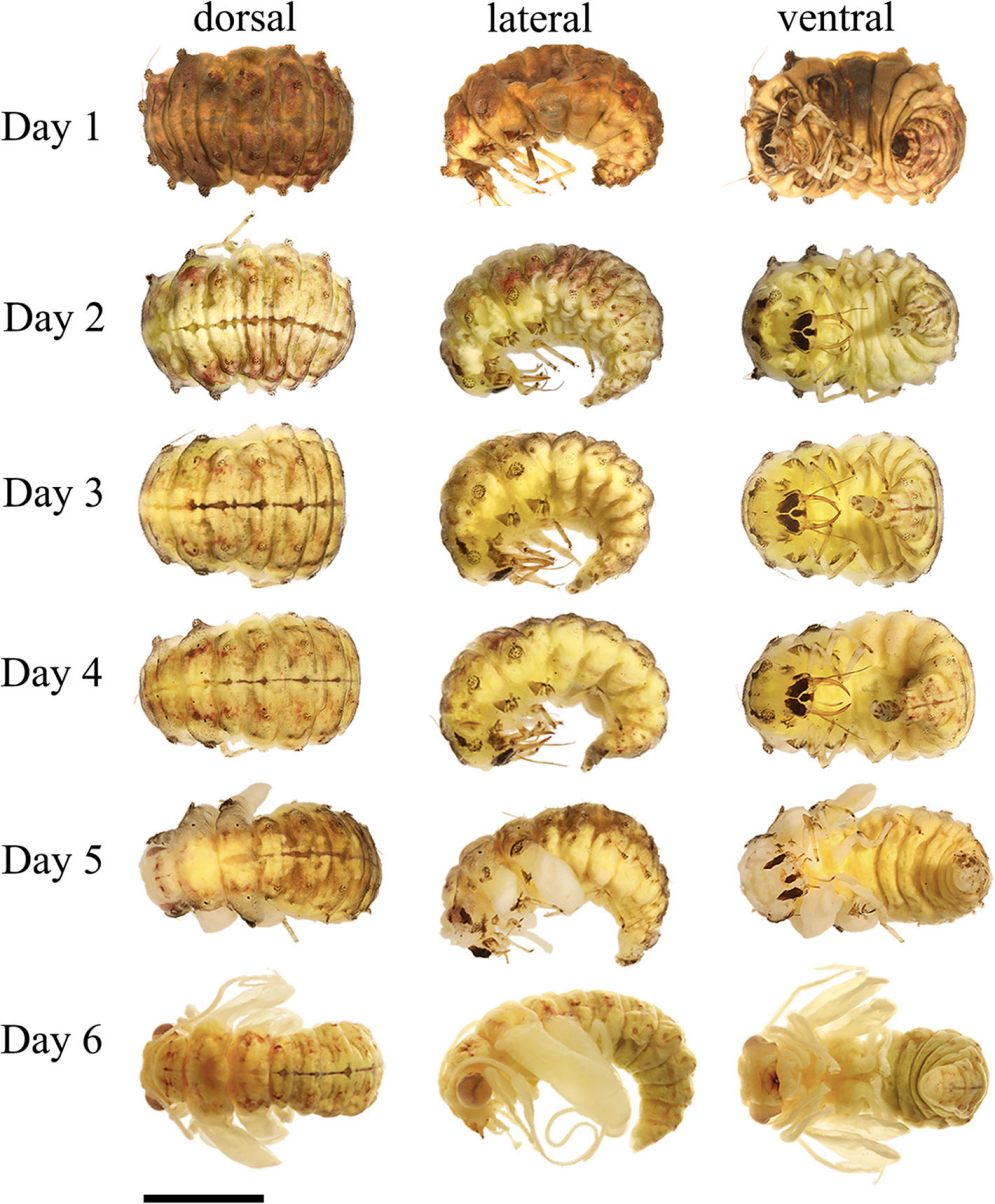
*Chrysopa pallens*, photographs: Day 1 to Day 6 after cocooning, dorsal, lateral, and ventral view. Scale bar: 2.5 mm

##### Pupae (Figs. 1b, Figs. 2-3). From day 5 to day 10

Color and body shape remains unchanged. At Day 5, larval cuticle cracked and wings present. Larval cuticle gathers under abdomen in cocoon (Fig. 1e). During pupal stage, two layers of cuticle are apparent, the very thin and transparent outer one is the pupal cuticle, and the inner one is the adult cuticle. Pharate adult 6.00 mm in length and 2.50 mm in height. Head 1.5 mm wide and 1.2 mm long. The head is very different from that of the larvae due to the hypognathous mouth parts. Compound eyes, scape, labrum, and mandible similar to adults. Color of labrum and mandibles turn red to crimson from Day 6 to Day 10. Compound eyes red to metallic black-red. Maxillary palps, labial palps, and curly antenna present with milky color. Frontoclypeal sulcus present. Wings become larger in size and folded in wing sheath. Prothorax, mesothorax, metathorax, and legs similar to adults in shape. Short setae present on frons. **Day 11:** Pharate adults develop well within pupal sheath, less sclerotized than adults. Wings brown to dark from base to distal margin distinctly. **Day 12:** Pupae break out from cocoon (Fig. 1f). After 3 h, they emerge (Fig. 1g).

**Fig. 3:**
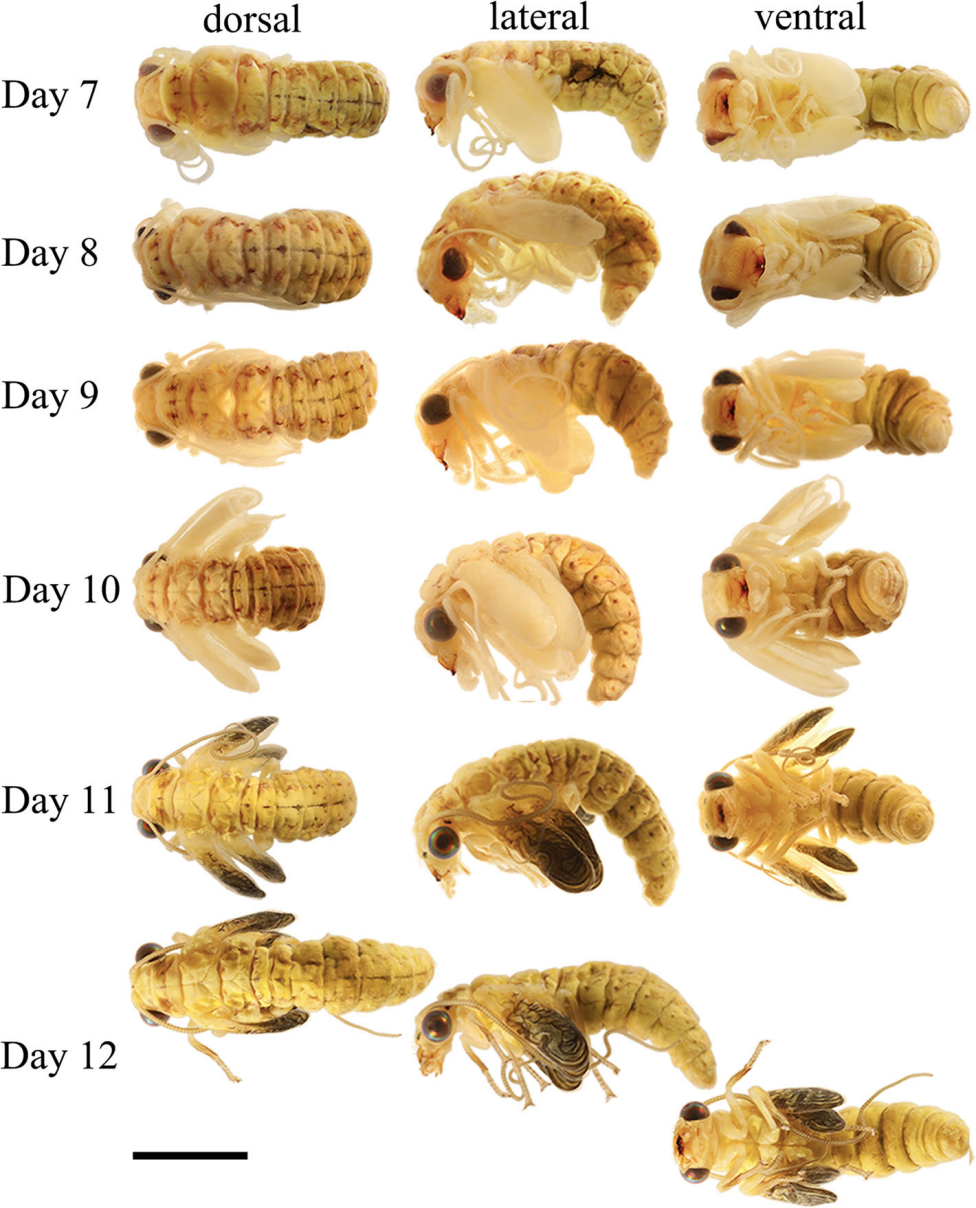
*Chrysopa pallens*, photographs: Day 7 to Day 12 after cocooning, dorsal, lateral, and ventral view. Scale bar: 2.5 mm

Adults (Fig. 1c). All structures well-developed, pale yellow. Adults 12.00 mm in length and 4.00 mm in height. Head 2.00 mm in width and 1.50 mm in length.

#### Head capsule

##### Third instar larvae (Fig. 4: a-c)

Head prognathous, roughly triangular, round posteriorly. Dorsum cream to light yellow with dark brown markings. Frontal markings confluent mesally, elongate. Epicranial markings paired, V-shape, not confluent mesally, extending to cervical margin. Eyes with six stemmata. Clypeus and labrum unmarked, continuous with frons. Membranous connection between labrum and clypeus completely reduced. Frontoclypeal sulcus absent. Anterior margin of head oblique in lateral view. Front region V-shape posteriorly and parallel-side anteriorly. Mandible amber, dark apically. Ventral maxilla smooth. The sucking tubes formed laterally by the interlocking of the mandibles and maxillae. Labium light brown. Gula absent.

**Fig. 4:**
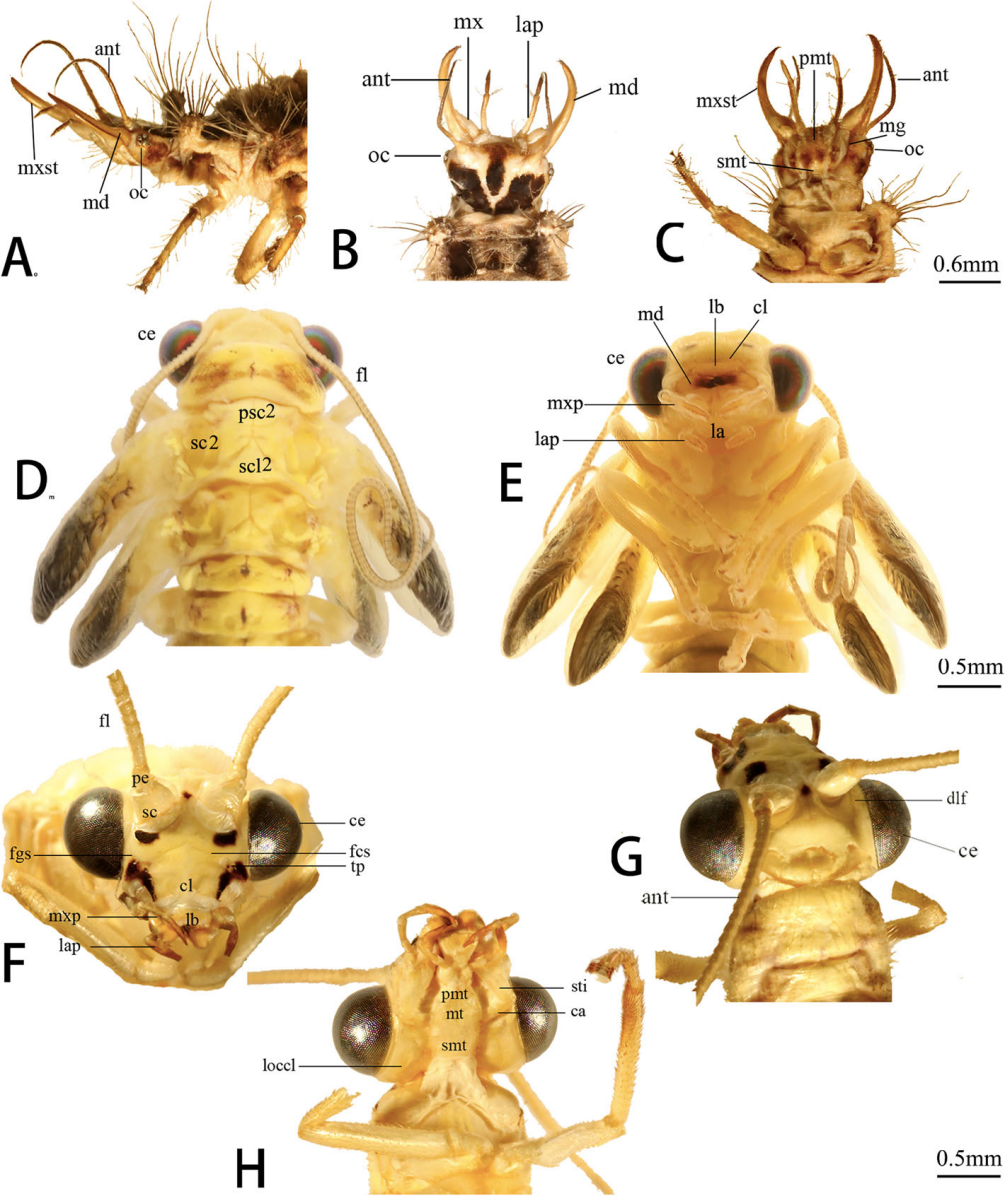
*Chrysopa pallens*, photographs: **a** larvae head, lateral view; **b** same, dorsal view; **c** same, ventral view; **d** pupae head of Day 11, dorsal view; **e** same, ventral view; **f** adults head, frontal view; **g** same, dorsal view; **h** same, ventral view. *Abbreviations*: ant: antenna; ca: cardo; ce: compound eye; cl: clypeus; dlf: dorsolateral longitudinal furrow; fcs: frontoclypeal sulcus; fgs: frontogenal suture; fl: flagellomeres; la: labium; lap: labial palp; lb.: labrum; loccl: lateral occipital lobes; md: mandible; mg: maxillary groove; mt: mentum; mx: maxilla; mxp: maxillary palp; mxst: maxillary stylet; oc: ocellus; pe: pedicellus; pmt.: prementum; psc2: mesothoracic prescutum; sc: scapus; sc2: mesothoracic scutum; scl2: mesothoracic scutellum; smt: submentum; sti: stipes; tp: tentorial pits

##### Pupae (Fig. 4: d-e). Day 11

Pharate adult head hypognathous, nearly triangular in frontal view, yellow to pale brown from vertex to mouthparts. Posterior vertex slightly concave. Compound eyes hemispherical, metallic black, occupying half of head width. Ocellus absent. Antennas locate between compound eyes. Antennomeres curly and almost one and a half times the body length, covering on sides of body. Clypeus broad. An indistinct suture present between clypeus and labrum. Lateral gena strongly round. Ventrally, labium connects with maxilla, which possesses 5-segments palpus.

##### Adults (Fig. 4: f-h)

Same shape and color to Day 11 pupae. Posterior vertex concave. Compound eyes large and metallic black, composed of numerous small and hexagonal ommatidia. Ocellus absent. Scapus swollen in antennal socket. Antenna filiform and almost as long as body length. Head nearly wedge-shaped in lateral view, gradually narrowing to mouthparts. Ecdysial line vestigial. Frontoclypeal sulcus and frontogenal suture present. Dorsolateral longitudinal furrow extends from dorsolateral margin of hind head capsule to mandible articulation. Lateral occipital lobes slightly exposed and hemispherical. Frontogenal suture connects anterior antennal fossa with dark anterior tentorial pits. Subgenal suture above mandible articulation vestigial. Lateral clypeus round. Anterior clypeus concave slightly with convex median line.

#### Tentorium

##### Third instar larvae (Figs. 5: larva in a, Fig. 6)

Tentorium fully sclerotized, tubular, solid throughout, connecting anterior tentorial pits at posterolateral clypeal margin with posterior tentorial pits at the foramen magnum. Tentorial bridge (tb) connects posterior tentorial arms (pta). Anterior tentorial arms (ata) diverge slightly. Dorsal tentorial arms (dta) well developed, attaching to head capsule directly.

**Fig. 5:**
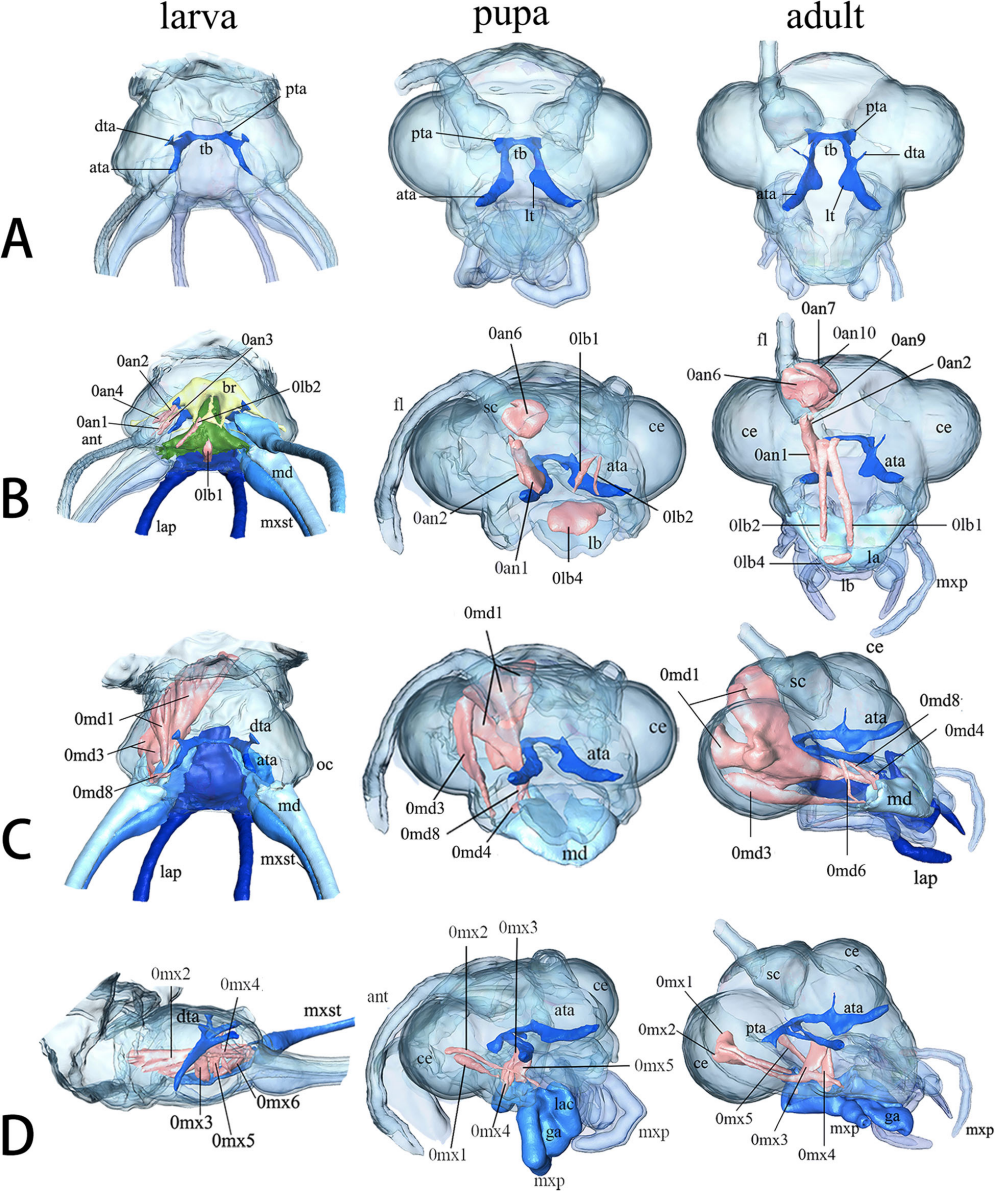
*Chrysopa pallens*, 3D reconstructions of head internal structures of larvae, pupae (Day 11), and adults, cuticle rendered transparent, muscles in light pink, brain in yellow, and pharynx in green: **a** tentorium, dorsal view; **b** half of labrum and antennal musculature, frontal view; **c** half of mandible musculature, frontal view; **d** half of maxillary musculature, lateral view. *Abbreviations*: ata: anterior tentorial arm; br: brain; ce: compound eye; dta: dorsal tentorial arm; fl: flagellomeres; ga: galea; la: labium; lac: lacinia; lap: labial palp; lb.: labrum; lt: laminatentorium; md: mandible; mxst: maxillary stylet; mxp: maxillary palp; oc: ocellus; pta: posterior tentorial arms; sc: scapus; tb: tentorial bridge

**Fig. 6:**
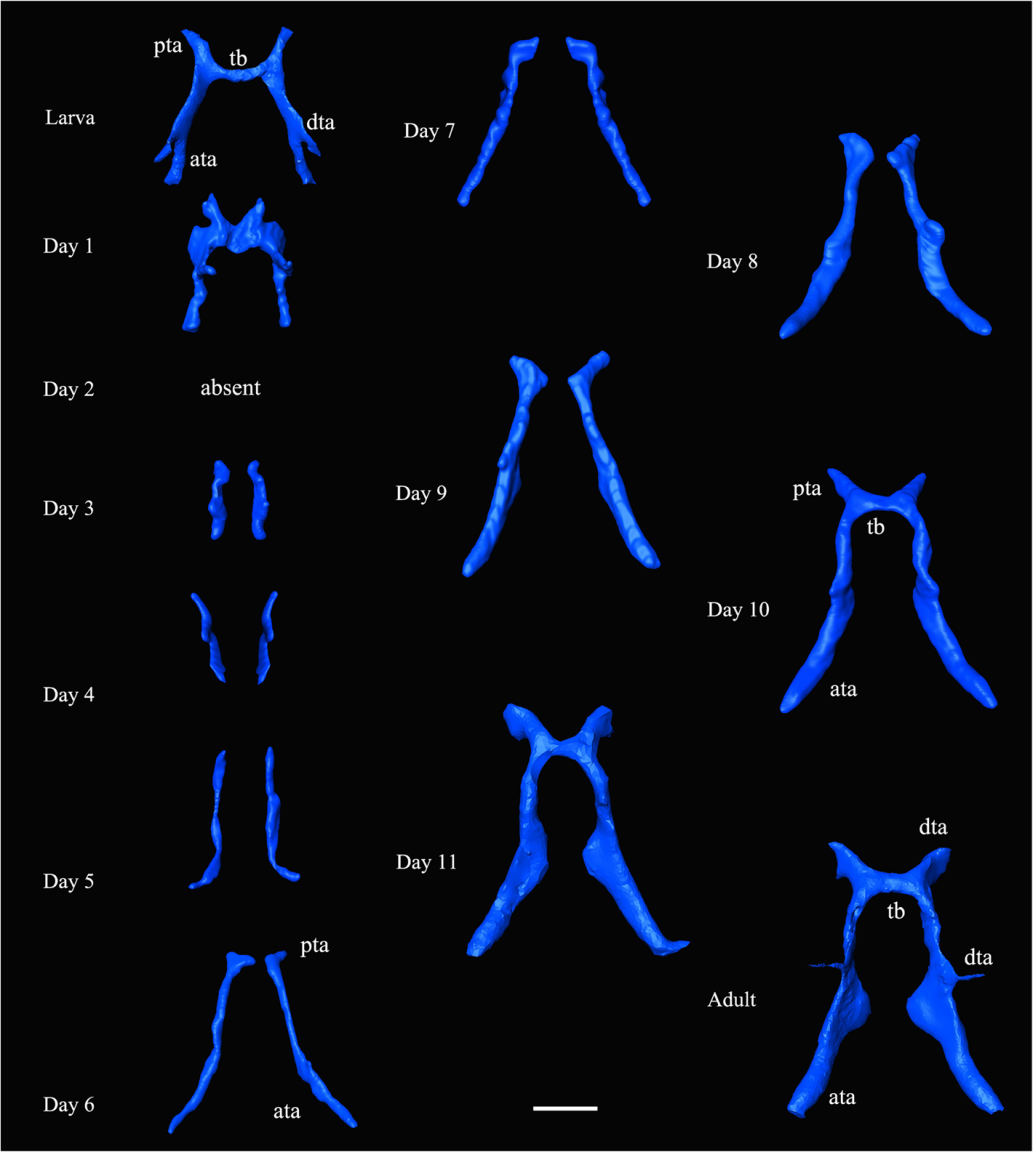
*Chrysopa pallens*, 3D reconstructions of tentorium from larvae to adults. Scale bar: 0.2 mm. *Abbreviations*: ata: anterior tentorial arm; dta: dorsal tentorial arm; pta: posterior tentorial arms; tb: tentorial bridge

##### Prepupae (Fig. 6)

At Day 1, ata, pta, and tb still exist, but tentorium dramatically compressed. By Day 2, tentorium disappeared. From Day 3, new tentorium present, including two separated arms.

##### Pupae (Figs. 5: pupa in a, Fig. 6)

Tentorium develop gradually at the following days. Boundary of ata and pta indistinct before Day 6. By Day 10, tb present. By Day 11, Tentorium sclerotized and hollow. Laminatentorium (lt) present which serves as attachment area of muscles (0an1, 0mx3, 0mx4, and 0mx5). Ata slender and diverge anteriorly.

##### Adults (Figs. 5: adult in a, Fig. 6)

Tentorium fully sclerotized, connecting larger anterior pits at posterolateral clypeal margin with posterior pits below occipital. Dta present, but very thin. Lt protruding, serving as the attachment of 0an1, 0mx3, and 0mx4.

#### Labrum

##### Third instar larvae (Fig. 4: a-c)

Labrum fused to clypeus but recognizable by slightly convex structure. Musculature: in Fig. 5: larvae in b.

##### Pupae (Fig. 4: d-e). Day 11

Pharate adult labrum dark brown and clypeus brown. Anterior labrum margin slightly convex. Anterolateral edges round. Musculature: in Fig. 5: pupa in b.

##### Adults (Fig. 4: g-h)

Labrum short, moving freely by labrum muscles. Anterior margin slightly convex. Two short tormae present on posterolateral labrum. Musculature: in Fig. 5: adult in b.

#### Antenna

##### Third instar larvae (Fig. 4: a-c)

Antenna glabrous and multisegmented in a slightly elevated socket. Basal segment globular and tapering distally. Pseudosegments cylindrical and separated indistinctly. Apical antennomere slender. Musculature: in Fig. 5: larva in B.

##### Pupae (Fig. 4: d-e). Day 11

Pharate adult antennae filiform and multisegmented, composed of a scapus, pedicellus and flagellomeres. Flagellomeres extremely elongate, about 1.5 times as long as pupal length, covering sides of thorax. Scapus proximally wide and narrow distally. Pedicellus nearly cylindrical with almost identical diameter and length. Musculature: in Fig. 5: pupa in b.

##### Adults (Fig. 4: g-h)

Antenna filiform, about 1/3 as long as forewing. Same location to pharate adult. Socket indistinct. Short setae present around each flagellomeres. Musculature: in Fig. 5: adult in b.

#### Mandible

##### Third instar larvae (Fig. 4: a-c)

Mandibles strongly elongate, slender with apical parts, slightly upturned, longer than labial palps, closely connected with elongate maxilla. Sucking channel enclosed by mandible and maxilla. Basal mandible wide. Apical mandibular stylet curved mesad and apically pointed. Mola, prostheca and subapical teeth absent. Mandibular surface smooth. Musculature: in Fig. 5: larva in c.

##### Pupae (Fig. 4: d-e, Fig. 7: pupa). Day 11

Pharate adult mandibles roughly triangular and not quite symmetric. Joints not clear. Upper surface convex and ventral concave. Both left and right mandibles possess three apical incisors. Molar process presents in middle region of mesal edge. Ventromesally, left molar concave to fit with convex right one. Musculature: in Fig. 5: pupa in C.

**Fig. 7:**
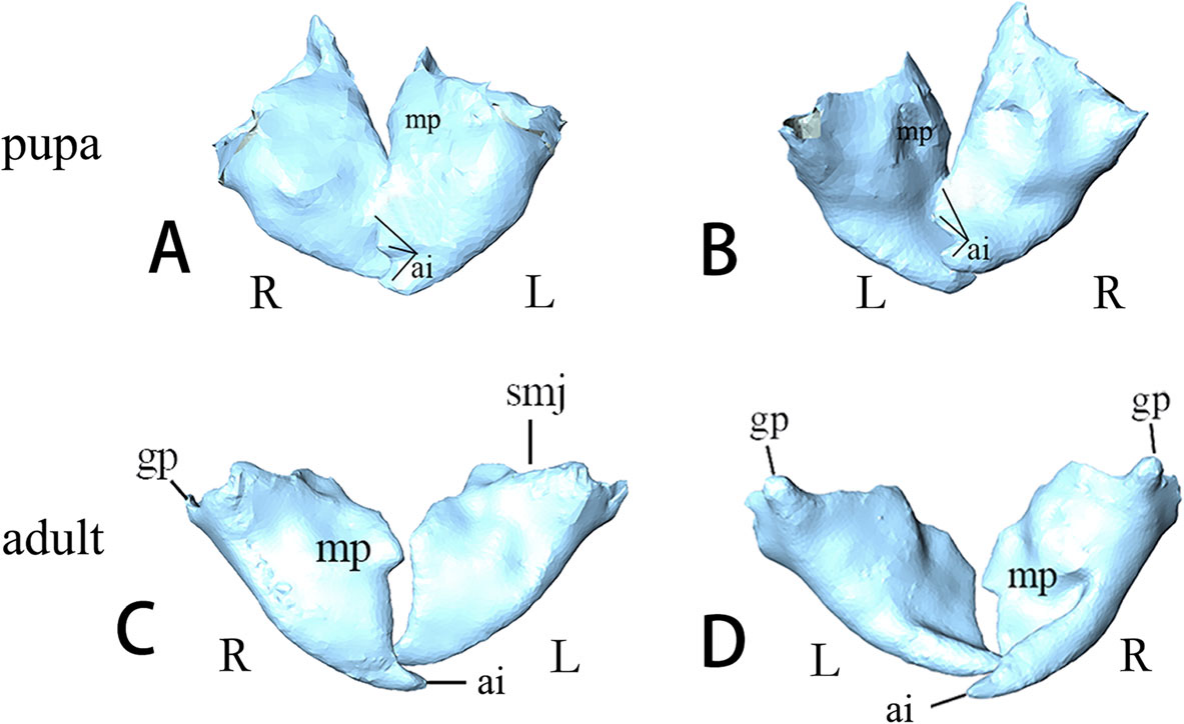
*Chrysopa pallens*, 3D reconstructions of mandibles: **a** mandible of pupae, dorsal view; **b** same, ventral view; **c** mandible of adults, dorsal view; **d** same, ventral view. *Abbreviations*: ai: apical incisor; gp: globular protrusion (primary mandiblular joint); L: left; mp: molar process; R: right; smj: secondary mandibular joint

##### Adults (Fig. 4: g-h, Fig. 7: adult)

Mandibles heavily sclerotized. Primary mandibular joint is a globular protrusion, articulated with shallow emargination of head capsule. Secondary mandibular joint formed by a cavity of mandible and a corresponding protrusion of head capsule. Left and right mandibles moderately asymmetric. Each has an apical incisor. Dorsal side slightly convex and ventral side moderately concave. Cutting edge nearly straight on left mandible but curved on right. Small triangular molar process present in middle region of mesal edge. It is more distinct on right than on left. Musculature: in Fig. 5: adult in c.

#### Maxilla

##### Third instar larvae (Fig. 4: a-c)

**Fig. 8:**
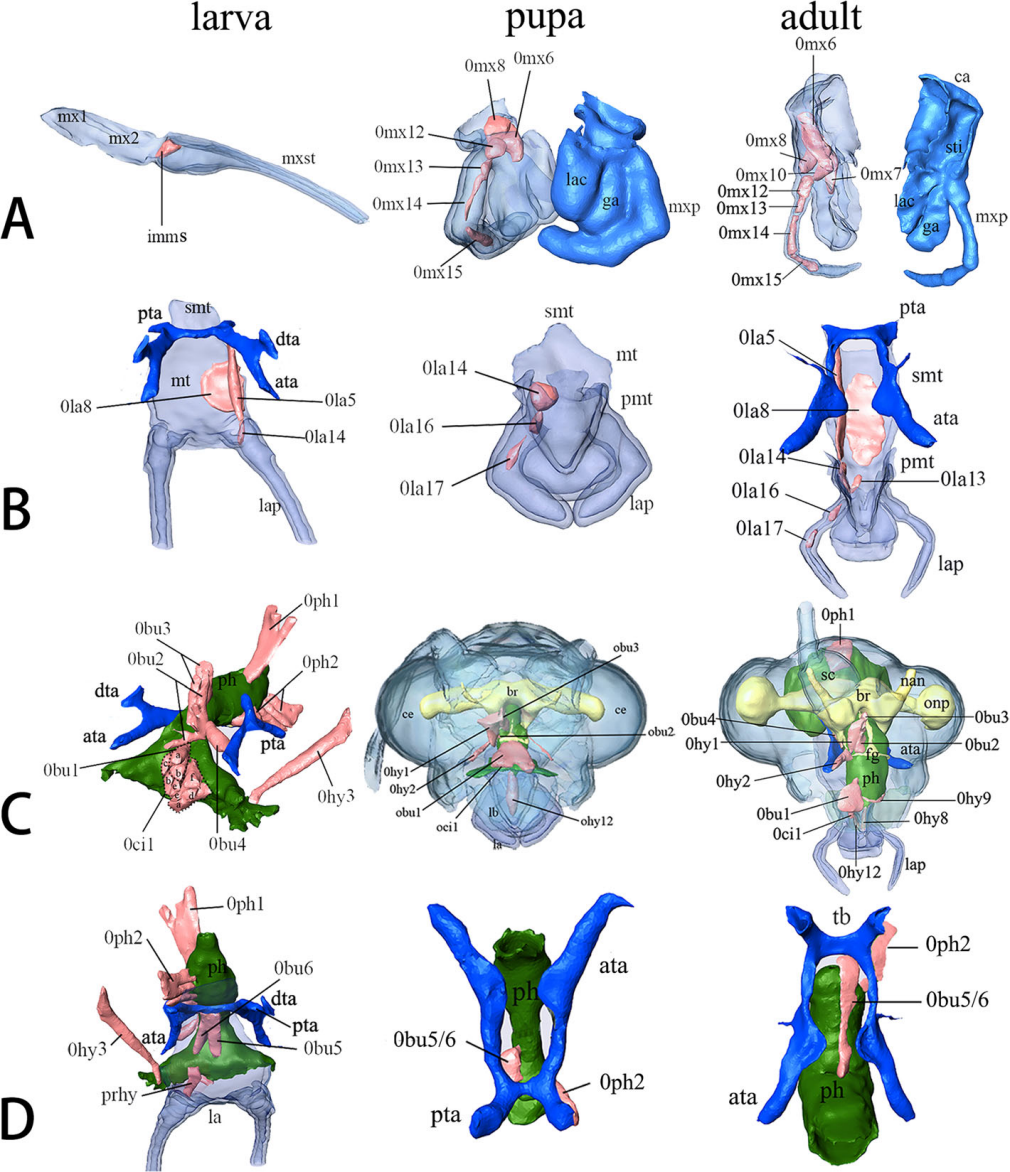
*Chrysopa pallens*, 3D reconstructions of head internal structures from larvae to adults: **a** maxilla musculature, dorsal view; **b** labium musculature, dorsal view; **c** musculature of epipharynx, pharynx, and hypopharynx, dorsal view; **d** musculature of hypopharynx and pharynx, ventral view. *Abbreviations*: ata: anterior tentorial arm; ant: antenna; br: brain; ca: cardo; ce: compound eye; dta: dorsal tentorial arm; fg: frontal ganglion; ga: galea; la: labium; lac; lacinia; lb.: labrum; imms, intrinsic muscle of maxillary stylet; lap: labial palp; md: mandible; mt: mentum; mx1: proximal maxillary element; mx2: intermediate maxillary element; mxp: maxillary palp; mxst: maxillary stylet; nan: antennal nerve; onp: optic neuropils; ph: pharynx; pmt.: prementum; prhy, prelabiohypopharyngeal muscle; pta: posterior tentorial arms; sc: scapus; smt: submentum; sog: suboesophageal ganglion; sti: stipes; tb: tentorial bridge

Maxilla composed of a proximal element, an intermediate part and an elongate distal maxillary stylet. Proximal element small, round laterally, oblique anteriorly. Intermediate piece larger, round laterally. A seta inserted in median region. Maxillary stylet elongates, similar to mandible in shape, forming the ventral part of sucking jaw. Apical part enfolds mandible. Musculature: in Fig. 5: larva in d, Fig. 8: larva in a.

##### Pupae (Fig. 4: d-e). Day 11

Pharate adult maxilla posterior to mandible. Cardo roughly quadrangular, broad. Stipes in similar shape with cardo and narrowing distally. 5-segments maxilla palpus insert on stipes distolaterally. Palpomere 1 shorter and broader. Palpomere 2 longer than 1 but wide distally. Three distal palpomeres slender. Palpomere 5 with a spindle-shaped apex. Proximal lacinia fused to dorsal stipes. Distal part slightly sickle-shaped. Galea slender proximally and wide distally, inserting between palp and lacinia. Musculature: in Fig. 5: pupa in d, Fig. 8: pupa in a.

##### Adults (Fig. 4: g-h)

Maxilla connects with submentum by membrane. Cardo roughly triangular. Stipes narrower and longer than cardo, forming an acute angle laterally at base. Palp inserts in lateral stipes. Palpomere 1 much broader than other 4 segments. Distal three palpomeres elongate and slender. Lacinia basally fused to dorsal stipes. Distal part slightly curved and sickle-shaped. Galea includes slender basigalea and broader distigalea. Musculature: in Figs. 5: adult in d, Fig. 8: adult in a.

#### Labium

##### Third instar larvae (Fig. 4: a-c)

Labium composed by submentum, mentum, and prementum, forming a complex with anterior hypopharynx. Submentum narrow and rectangular, laterally connecting with cardo. Anterior edge separated from mentum by distinct convex. Anterior mentum flat, wide, round anterolaterally. Two pairs of setae insert at anterior mentum. Prementum small and medially divided by a cleft. Glossae, paraglossae, and ligula absent. 3-segments palp (lap) distinctly elongate. Basal segment cylindrical. Palpomere 2 extremely elongate, about ten times as long as wide and slightly wide distally. Palpomere 3 slender, with same length to palpomere 1. Musculature: in Fig. 8: larva in b.

##### Pupae (Fig. 4: d-e). Day 11

Pharate adult submentum short and narrow, separated by mentum by suddenly wide anterior margin. Mentum flat and slightly swollen. Prementum carries ligula with 3-segments palp. Palpomere 3 longer than palpomere 1 and 2. Ligula diamond-shaped and sclerotized. Musculature: in Fig. 8: pupa in b.

##### Adults (Fig. 4: g-h)

Elemental composition stays same to Day 11 pharate adult. Submentum edge not clear, recognized by muscles attachment. Labium possess well developed 3-segments palps. Ligula large and sclerotized with paired paraglossae. Musculature: in Fig. 8: adult in b.

#### Epipharynx

##### Third instar larvae

Epipharynx, ventral surface of anterior clypeolabrum, sclerotized and slightly convex. Posterior membranous epipharynx fused to anterior pharynx and posterior hypopharynx laterally, forming the dorsal part of the closed prepharygeal tube. Musculature: in Fig. 8: larva in c.

##### Pupae. Day 11

Pharate adult anterior epipharynx membranous, covering basal mandible. Posterior epipharynx and hypopharynx fused to anterior pharynx margin, forming anterior pharynx. Musculature: in Fig. 8: pupa in c.

##### Adults

Same to pharate adult in Day 11. Musculature: in Fig. 8: adult in c.

#### Hypopharynx and salivarium

##### Third instar larvae

Anterior hypopharynx closely connected with anterior labium. Weak sclerotized above prementum and mentum. Posterior hypopharynx laterally fused to posterior epipharynx, forming the ventral prepharygeal tube. Salivarium absent. Musculature: in Fig. 8: larva in c-d.

##### Pupae. Day 11

Pharate adult hypopharynx not fully developed, fused to ventral pharynx. Salivarium and salivary duct not well-developed. Musculature: in Fig. 8: pupa in c.

##### Adults

Hypopharynx forms a structural and functional unit with anterior labium. Anterior part extends to ligula. Dorsolaterally, oral arms slender and run along hypopharynx. Hypopharyngeal suspensorial sclerites forms lateral short branch, closely connected with ventral ridge of prementum. Salivary duct broad and quardrangular in cross section above submentum and mentum. Musculature: in Fig. 8: adult in c.

#### Pharynx

##### Third instar larvae (Fig. 8: larva in c-d)

Anterior precerebral pharynx V-shape. Following region approximately quadrangular in cross section with indistinct longitudinal folds for muscles attachment. Protocerebrum pharynx gradually narrow distally and irregular in cross section. Musculature: in Fig. 8: larva in c-d.

##### Pupae (Fig. 8: pupa in c-d). Day 11

Pharate adult pharynx narrow especially beneath brain. Precerebral pharynx slightly wide anteriorly. Cross section nearly oval. Pharynx wall thick and longitudinal folds indistinct. Postcerebral pharynx narrow. Musculature: in Fig. 8: pupa in c-d.

##### Adults (Fig. 8: adult in c-d)

Anterior precerebral pharynx wide and nearly round in cross section. Pharynx wall thin and no distinct longitudinal folds. Postcerebral pharynx suddenly wide with thick wall. Longitudinal folds present. Musculature: in Fig. 8: adult in c-d.

#### Transformation of muscles inside cocoon

**Fig. 9:**
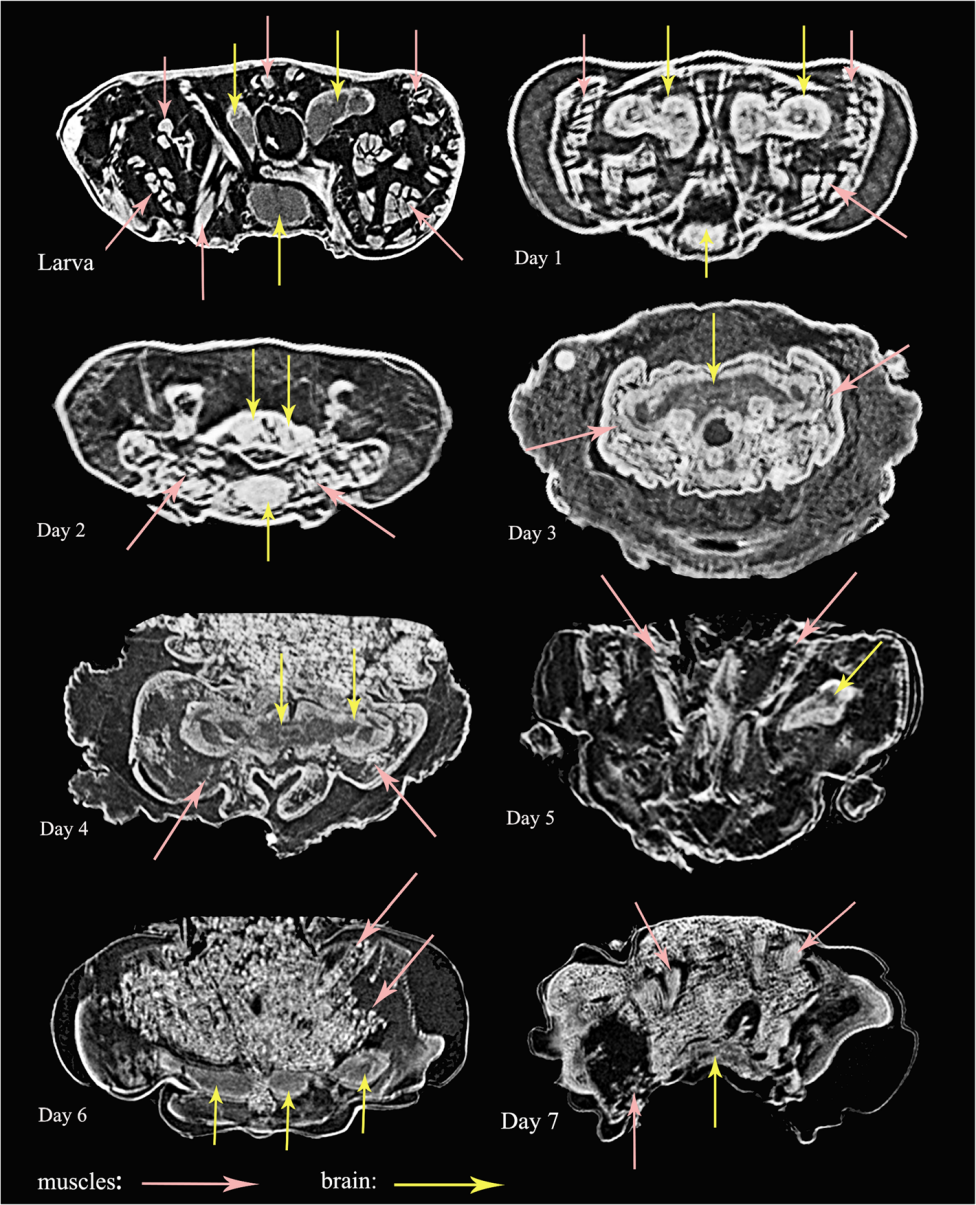
*Chrysopa pallens*, cross sections from micro-CT of head from larvae to pupae. Muscle fibers in pink arrow, nerves in yellow arrow

**Fig. 10:**
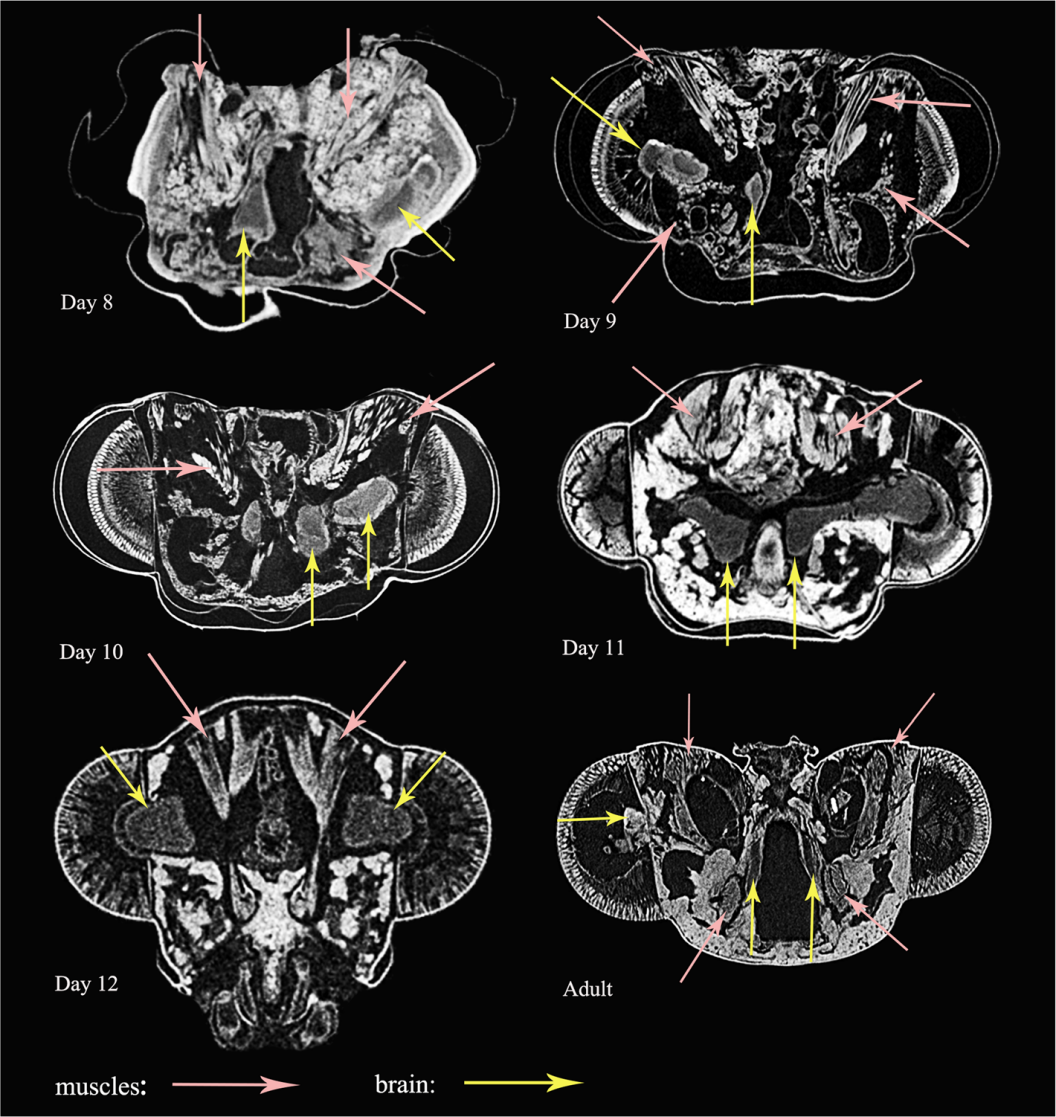
*Chrysopa pallens*, cross sections from micro-CT of head from pupae to adults. Muscle fibers in pink arrow, nerves in yellow arrow

**Fig. 11:**
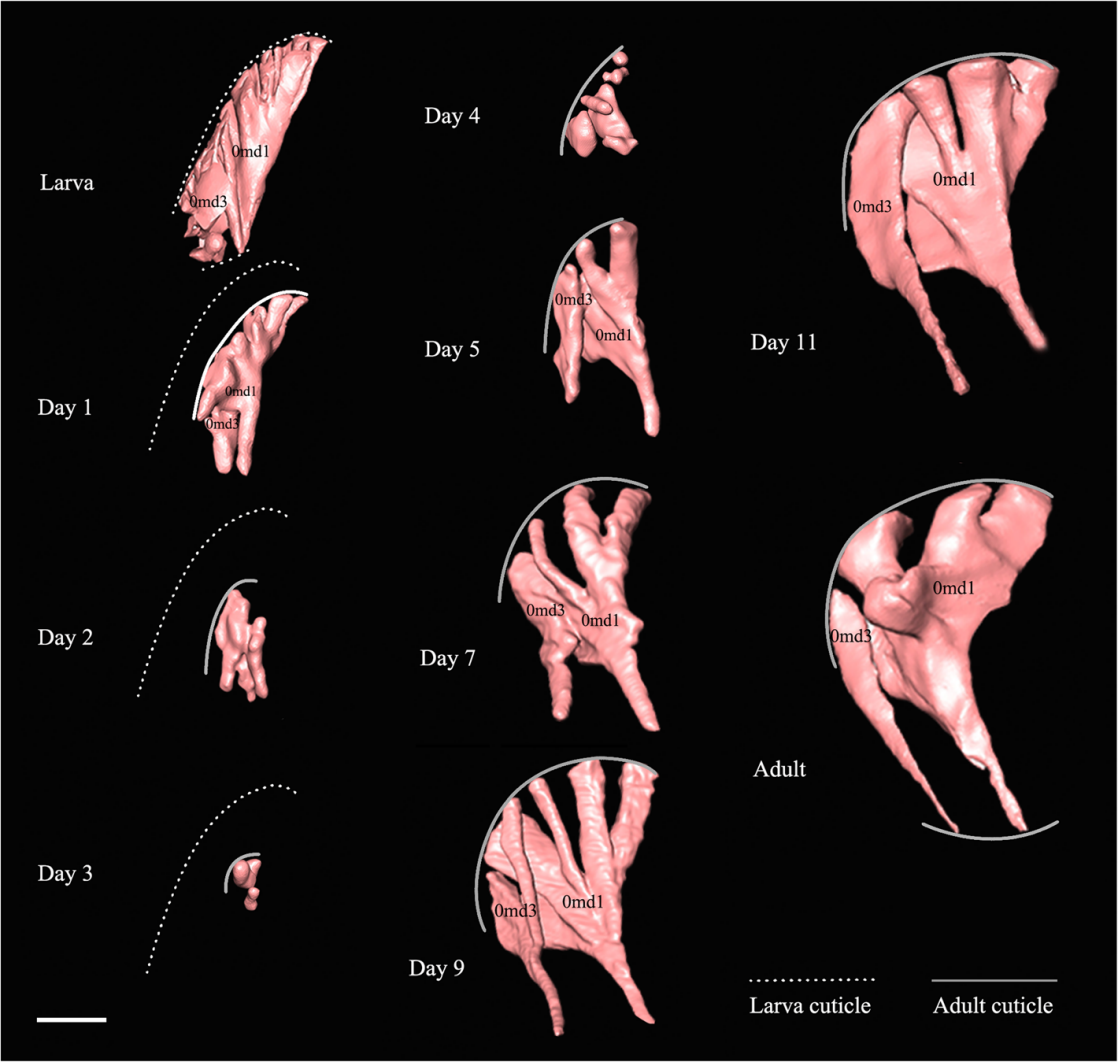
*Chrysopa pallens*, 3D reconstructions of mandible muscles (0md1, 0md3) from larvae to adults. Muscles in pink. The larval cuticle is represented by dotted line and adult cuticle is represented by solid line. Scale bar: 0.15 mm

The transformation of head muscles from Day 1 to Day 12 are illustrated in Figs. 9-10. The histolysis and rebuilding of the skeleton and muscles happens in the prepupal stage, with these structures developing gradually in the following days until the adult stage. For example, the mandible muscles 0md1 and 0md3 are reconstructed in detail in Fig. 11. At Day 1, muscles are compressed by the inner cuticle. By Day 2, the inner cuticle is strongly compressed, and most muscles have disintegrated. New skeletal structures begin to form. At Day 3, remaining muscle tissues disintegrate continuously. By Day 4, new muscle granules are present. At Day 5, muscle fibers are present. More and more muscle fibers and bundles present in the following days. By Day 12, almost all muscles present in bundle form.

### Cephalic nervous system

The main elements of the central nervous system are the brain and the subesophageal ganglion. The latter is the first ganglion of ventral nerve cord. The two with the frontal ganglion are the main elements of the cephalic nervous system.

#### Cerebrum, suboesophageal complex, and frontal ganglion

##### Third instar larvae (Fig. 12: larva)

Size of brain and suboesophageal ganglion (sog) about 20% that of the entire head capsule. Brain composed of the protocerebrum, deutocerebrum, and tritocerebrum. Protocerebrum dumbbell-shaped and optical nerves extremely slender with very slightly round lobe. Two thin antennal nerves originate from slightly protruding region of deutocerebrum. Frontal connectives originate from tritocerebrum and circumoesophageal connectives continuous with tritocerebrum. Sog ovoid-shaped below pharynx. All slender nerves of labium, maxilla, and mandible originate from sog. Frontal ganglion triangular, connecting with the protocerebrum and tritocerebrum by three curved frontal connectives.

**Fig. 12:**
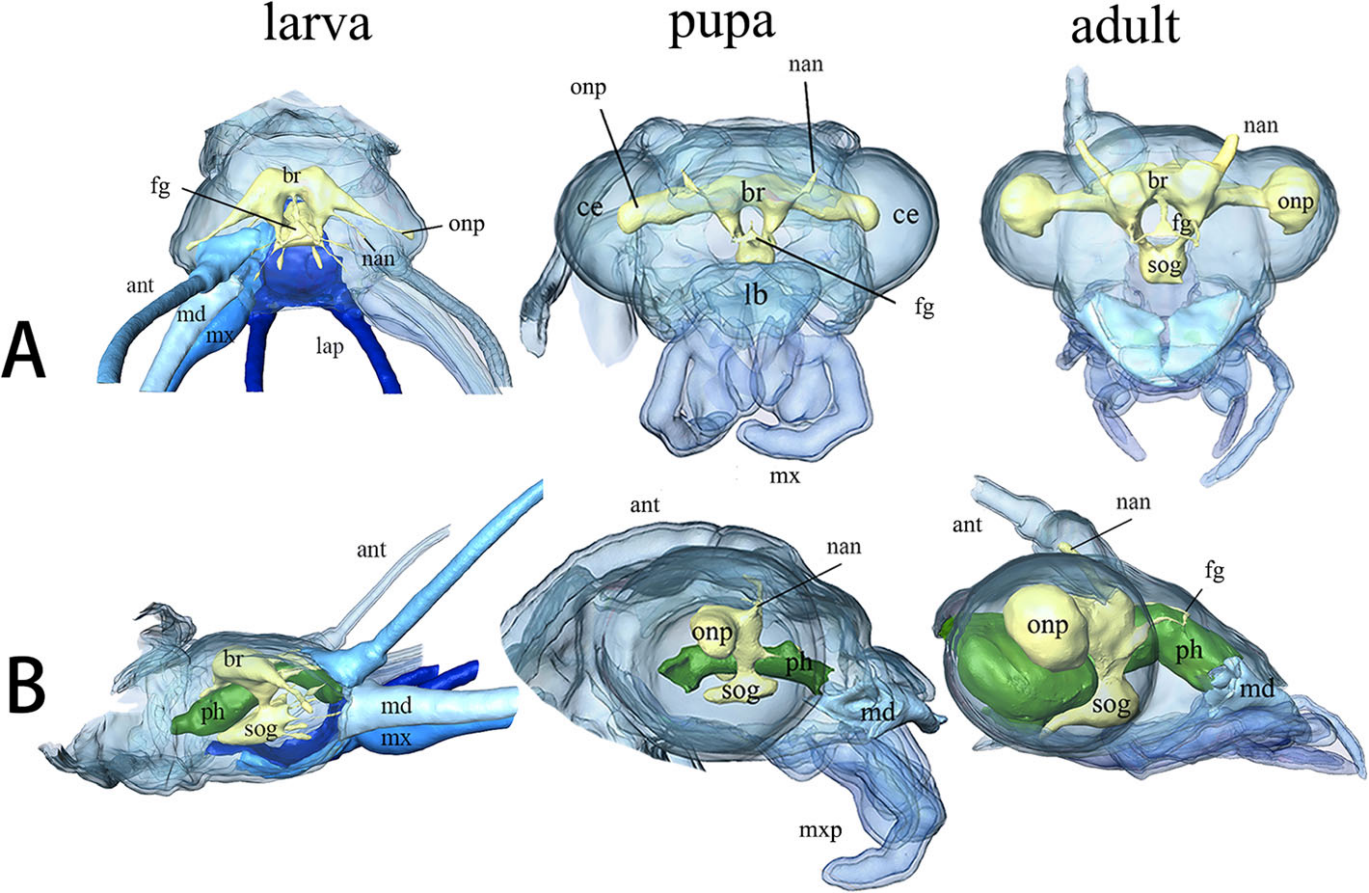
*Chrysopa pallens*, 3D reconstructions of cephalic nervous system from larvae to adults: **a** brain and suboesophageal ganglion, dorsal view; **b** same, lateral view. *Abbreviations*: ant: antenna; br: brain; ce: compound eye; fg: frontal ganglion; lb.: labrum; lap: labial palp; md: mandible; mx: maxilla; mxp: maxillary palp; nan: antennal nerve; onp: optic neuropils; ph: pharynx sog: suboesophageal ganglion

##### Pupae (Fig. 12: pupa). Day 11

Pharate adult volume of brain and suboesophageal complex small, occupying about 12.5% that of head capsule. Protocerebrum unrepresentative of dumbbell-shape. Optical nerves cylindrical with slightly round lobe. Antennal nerves slender and bending upwards. Tritocerebrum bears circumoesophageal connectives. Suboesophageal complex nearly oval. Frontal ganglion triangular and connected by two curved frontal connectives.

##### Adults (Fig. 12: adult)

Volume of brain and suboesophageal complex occupies about 33.3% that of head capsule. Protocerebrum dumbbell-shaped with two large optic neuropils. Suboesophageal complex oval. Triangular frontal ganglion connected by three nerves like larvae.

#### Transformation of brains inside cocoon

**Fig. 13:**
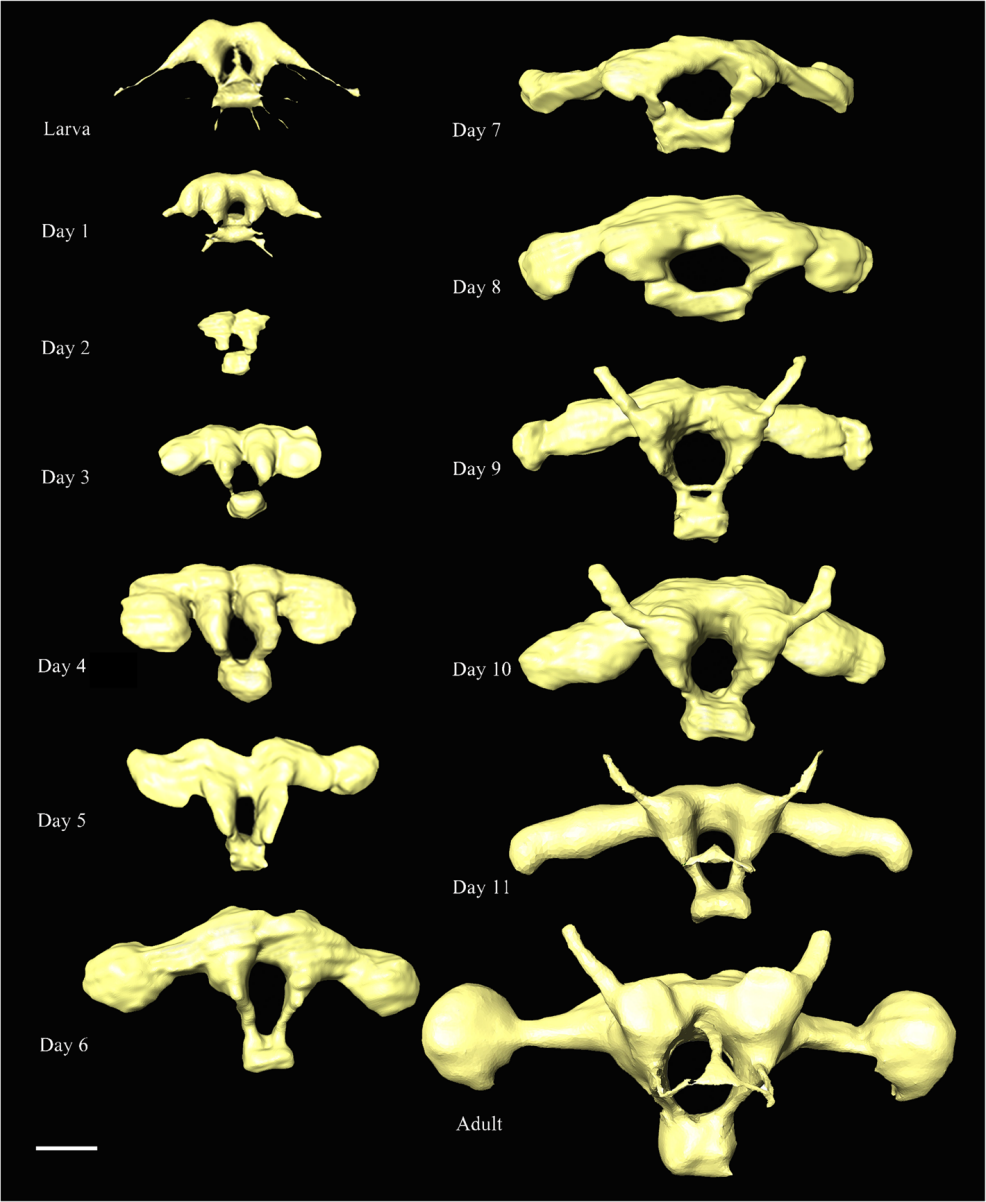
*Chrysopa pallens*, 3D reconstructions of brain from larvae to adults in front view. Scale bar: 0.2 mm

Transformation of brains from Day 1 to Day 12 is illustrated in Figs. 9-10, 13. The larval brain does not disappear completely, but there is a morphological change. At Day 1, the brain becomes small and simple. Antennal nerves, optical neuropils, and mouthparts nerves distinctly short. Frontal ganglion disintegrated. By Day 2, brain strongly compressed and suboesophageal ganglion separated from brain due to disappearance of circumoesophageal connectives. From Day 3, brain stops compression but becomes more and more larger over following days. By Day 9, slender antennal nerves present. By Day 11, frontal ganglion present.

## Discussion

### Developmental transformations in larvae and adults

Niche differentiation of larvae and adults is often considered one important factor for the unparalleled evolutionary success of Holometabola [2, 37], but the differentiation is poorly developed in some taxa. A good example is *Chrysopa pallens* which utilizes the same diet (aphids) as larvae and adults with both stages having a similar competency when it comes to predation. The fact that all postembryonic stages of *Chrysopa pallens* are predatory is one of the reasons why this species is so popular as a biological control agent for aphids. In other green lacewing species, the larvae are predators while the adults mainly feed on nectar and honey dew. Predatory larvae and adults also make *C. pallens* an attractive target for our study of the development of head morphology because diet differences are unlikely to explain the morphological differences between larval and adult mouthparts. Yet, they differ significantly. Nearly all character systems are affected although many cephalic muscles can still be homologized (see Table 4 and Table 5). Many of the differences between the larval and adult stages are likely explained by the need of the adults to disperse and reproduce. The most striking differences are the better developed sensory organs; i.e., longer antennae, more sensillae, and compound eyes. Not surprisingly, the brain of the adults is also much larger.

**Table 4:** Homologization of the cephalic musculature of *Chrysopa pallens* (Rambur, 1838) from larvae to adults with the terminology of Wipfler et al. (2011) [36]

Muscle name	Abb.	Larvae	Pupae	Adults	Presumed function
Labrum					
M. frontolabralis	0 lb1	+	+	+	levator of labrum
M. frontoepipharyngalis	0 lb2	+	+	+	levator of labrum
M. labralis transversalis	0 lb4	–	+	+	compressor of labrum
Antenna					
M. tentorioscapalis anterior	0an1	+	+	+	depressor and flexor of antenna
M. tentorioscapalis posterior	0an2	+	+	+	levator of antenna
M. tentorioscapalis lateralis	0an3	+	–	–	depressor and rotator of antenna
M. tentorioscapalis medialis	0an4	+	–	+	depressor and rotator of antenna
M. scapopedicellaris lateralis	0an6	–	+	+	extensor of flagellum
M. scapopedicellaris medialis	0an7	–	+	+	flexor of flagellum
M. scapopedicellaris posterior	0an9	–	–	+	depressor of the antenna
M. scapopedicellaris anterior	0an10	–	–	+	elevator of the antenna
Mandible					
M. craniomandibularis internus	0md1	+	+	+	adductor of mandible
M. craniomandibularis externus posterior	0md3	+	+	+	abductor of mandible
M. hypopharyngomandibularis	0md4	–	+	+	protractor of anatomical mouth opening,
M. tentoriomandibularis lateral inferior	0md6	–	–	+	adductor of mandible
M. tentoriomandibularis medialis inferior	0md8	+	+	+	adductor of mandible
Maxilla					
M. craniocardinalis	0mx1	–	+	+	promoter of maxilla
M. craniolacinialis	0mx2	+	+	+	adductor of lacinia
M. tentoriocardinalis	0mx3	+	+	+	adductor of cardo and protractor of maxilla
M. tentoriostipitalis anterior	0mx4	+	+	+	adductor of maxilla
M. tentoriostipitalis posterior	0mx5	+	+	+	adductor of stipes and protractor of maxilla
M. stipitolacinialis	0mx6	+	+	+	adductor of lacinia
M. stipitogalealis	0mx7	–	–	+	abductor of galea
M. stipitopalpalis externus	0mx8	–	+	+	abductor of maxillary palp
M. stipitopalpalis internus	0mx10	–	–	+	adductor of maxillary palp
M. palpopalpalismaxillae primus	0mx12	–	+	+	adductor of maxillary palpomere ii
M. palpopalpalismaxillae secundus	0mx13	–	+	+	abductor of maxillary palpomere iii
M. palpopalpalismaxillae tertius	0mx14	–	+	+	adductor of maxillary palpomere iv
M. palpopalpalismaxillae quartus	0mx15	–	+	+	adductor of maxillary palpomere v
M. intrinsic muscle of maxillary stylet	imm	+	–	–	
Labium					
M. tentoriopraementalis	0la5	+	–	+	adductor of praementum
M. submentopraementalis	0la8	+	–	+	retractor of praementum
M. praementopalpalis internus	0la13	–	–	+	adductor of labial palpomere i
M. praementopalpalis externus	0la14	+	+	+	levator of labial palp
M. palpopalpalislabii primus	0la16	–	+	+	flexor of labial palpomere ii
M. palpopalpalislabii secundus	0la17	–	+	+	flexor of labial palpomere iii
Epipharynx					
M. clypeopalatalis	0ci1	+	+	+	dilatator ofcibarium
M. clypeobuccalis	0bu1	+	+	+	dilator of buccal cavity
Hypopharynx					
M. frontooralis	0hy1	–	+	+	
M. tentoriooralis	0hy2	–	+	+	
M. craniohypopharyngalis	0hy3	+	–	–	
M. praementosalivaris posterior	0hy8	–	–	+	
M. oralis transversalis	0hy9	–	–	+	
M. hypopharyngosalivaris	0hy12	–	+	+	dilator of salivarium
Pharynx					
M. frontobuccalis anterior	0bu2	+	+	+	dilator of pharynx
M. frontobuccalis posterior	0bu3	+	+	+	dilator of pharynx
M. tentoriobuccalis lateralis posterior	0bu4	+	–	+	
M. tentoriobuccalis anterior	0bu5	+	+	+	dilator of pharynx
M. tentoriobuccalis posterior	0bu6	+	+	+	dilator of pharynx
M. prelabiohypopharyngeal muscle	prhy	+	–	–	dilator of pharynx
M. verticopharyngealis	0 ph 1	+	–	+	dilator of pharynx
M. tentoriopharyngealis	0 ph 2	+	+	+	dilator of pharynx
*M. annularis* stomodaei	0st1	+	–	+	constrictor of the pharynx
M. longitudinalis stomodaei	0st2	+	–	+	constrictor of the pharynx

“+” = present, “-” = absent

**Table 5:** Presumed homologies of the cephalic muscles of *Chrysopa pallens* (Rambur, 1838) with muscles reported in von Kéler (1963) [38] and Miller (1933) [35]

Muscle name	Abb.	Present study	Kéler (1963) [38]	Mille (1933) [35]
Labrum				
M. frontolabralis	0 lb1	+	M8	1
M. frontoepipharyngalis	0 lb2	+	M9	2
M. labralis transversalis	0 lb4	+	–	3
Antenna				
M. tentorioscapalis anterior	0an1	+	M1	27
M. tentorioscapalis posterior	0an2	+	M2	28
M. tentorioscapalis lateralis	0an3	–	M3	–
M. tentorioscapalis medialis	0an4	+	M4	–
M. scapopedicellaris lateralis	0an6	+	M5	29
M. scapopedicellaris medialis	0an7	+	M6	30
M. scapopedicellaris posterior	0an9	+	–	32
M. scapopedicellaris anterior	0an10	+	–	31
Mandible				
M. craniomandibularis internus	0md1	+	M11	5–1
M. craniomandibularis externus posterior	0md3	+	M12	4
M. hypopharyngomandibularis	0md4	+	M13	5–2
M. tentoriomandibularis lateral inferior	0md6	+	–	–
M. tentoriomandibularis medialis inferior	0md8	+	–	–
Maxilla				
M. craniocardinalis	0mx1	+	M15	6
M. craniolacinialis	0mx2	+	M19	10
M. tentoriocardinalis	0mx3	+	M17	7b
M. tentoriostipitalis anterior	0mx4	+	M18	8
M. tentoriostipitalis posterior	0mx5	+	–	9
M. stipitolacinialis	0mx6	+	M20	13
M. stipitogalealis	0mx7	+	M21	14
M. stipitopalpalis externus	0mx8	+	M22	11
M. stipitopalpalis internus	0mx10	+	M23	12
M. palpopalpalismaxillae primus	0mx12	+	M24	15
M. palpopalpalismaxillae secundus	0mx13	+	M25	16
M. palpopalpalismaxillae tertius	0mx14	+	M26	17
M. palpopalpalismaxillae quartus	0mx15	+	M27	18
Labium				
M. tentoriopraementalis	0la5	+	M29	23/24
M. submentopraementalis	0la8	+	M28	22
M. praementopalpalis internus	0la13	+	M33	20
M. praementopalpalis externus	0la14	+	M34	19
M. palpopalpalislabii primus	0la16	+	M35	25
M. palpopalpalislabii secundus	0la17	+	M36	26
Epipharynx				
M. clypeopalatalis	0ci1	+	M43	–
M. clypeobuccalis	0bu1	+	M44	38
Hypopharynx				
M. frontooralis	0hy1	+	M41a	38
M. tentoriooralis	0hy2	+	M41b	42
M. tentoriohypopharyngealis	0hy3	+	M42	36
M. praementosalivaris posterior	0hy8	+	M39	33
M. oralis transversalis	0hy9	+	M67	–
M. hypopharyngosalivaris	0hy12	+	M37	34
Pharynx				
M. frontobuccalis anterior	0bu2	+	M45	39
M. frontobuccalis posterior	0bu3	+	M46	40
M. tentoriobuccalis lateralis posterior	0bu4	+	M49	43
M. tentoriobuccalis anterior	0bu5	+	M48	44
M. tentoriobuccalis posterior	0bu6	+	M50	45
M. verticopharyngealis	0 ph 1	+	M51	41
M. tentoriopharyngealis	0 ph 2	+	M52	46

“+” = present, “-” = absent

The most conspicuous change during metamorphosis is the orientation of the head and the morphology of the mouth parts. The larvae are prognathous with sucking tubes while the adults are hypognathous and have chewing mouthparts. Functionally, this appears to impact midgut morphology. In larvae, the midgut is discontinuous with the hind gut, and the solid waste is not passed until the adult emerges from the pupal case with a fully formed digestive system [39]. It appears that sucking liquefied prey allows the larvae to gain more energy with a shorter digestive tract. Furthermore, the prognathous head presumably helps with attacking aphids because the sucking mouthparts touch the aphids first and can function as a sword. Although predation is no longer the main task of adults, chewing mouthparts enables them to also prey quickly and efficiently. It is conceivable that the hypognathous mouthparts are more suitable for flying adults, but it is certain that the downward shift of the mouthparts from larvae to adults impacts many other aspects of head morphology. This includes the concave submentum and broader vertex of the adults. Additionally, the wedge-shaped head capsule of the adult has to accommodate the modified mouthparts, larger cephalic nervous system, and a strong musculature (e.g., 0an1 and 0an2). For most holometabolous insects, the upward or downward changes in the orientation of mouthparts between the larval and adult stage require some of the most dramatic changes within the pupae (see also the upward orientation of mouthparts in some Coleoptera: [14]).

The necessity to find a potential mating partner requires a far more complicated sensory system in the adults than is present in larvae: instead of simple stemmata, the adults have compound eyes. This change of the visual system requires a major modification of the brain, notably in the optic lobes which greatly increase in size. Similarly, the antennae are greatly elongated in adults. The antennal nerves also become larger in size than what is observed in the previous stages. In order to execute controlled movements of the adult antennae, a more complex muscle system is required. Three extrinsic and four intrinsic muscles are present in the antennae of adults, whereas only four small extrinsic muscles and no intrinsic muscle are found in larvae. It is conceivable that the intrinsic muscles allow for more effective control during flight than the extrinsic muscles. Additionally, from larvae to adults, the number of segments of the labial palp is constant but two additional intrinsic and one additional extrinsic muscle are present in adults. The Maxillary palps is absent in larvae, but the adults have a five-segmented palp that can be moved using four intrinsic and two extrinsic muscles.

In addition to the modifications mentioned above, an intrinsic muscle of maxillary stylet (imms) connecting the dorsal wall and ventral wall of stylet is present in larvae but absent in adults. This muscle is also known from the larvae of the neuropteran *Nevrorthus* [40]. Functionally, it probably controls the movement of the stylet and is one of several features that renders the larvae of Neuroptera morpho-larvae sensu stricto. Another feature is the sucking mouthparts whose channel can be modified via muscular contraction. This presumably assists with the pumping motion that is needed for injecting poison and sucking out liquefied prey.

The pharynx musculature consists of ten muscle bundles in larvae and nine in adults. The only muscle missing in adults is the one known as M. prelabiohypopharyngeal (prhy) in larvae. It is also known from the neuropteran larvae of *Nevrorthus* [40]. The muscle maybe needed for ensuring the stability of the labium and anterior pharynx in larvae. However, this muscle may limit the movement of labium in adults where more flexibility may be needed for manipulating prey. This may explain why it is lost during metamorphosis.

### Transformations inside cocoon

**Fig. 14:**
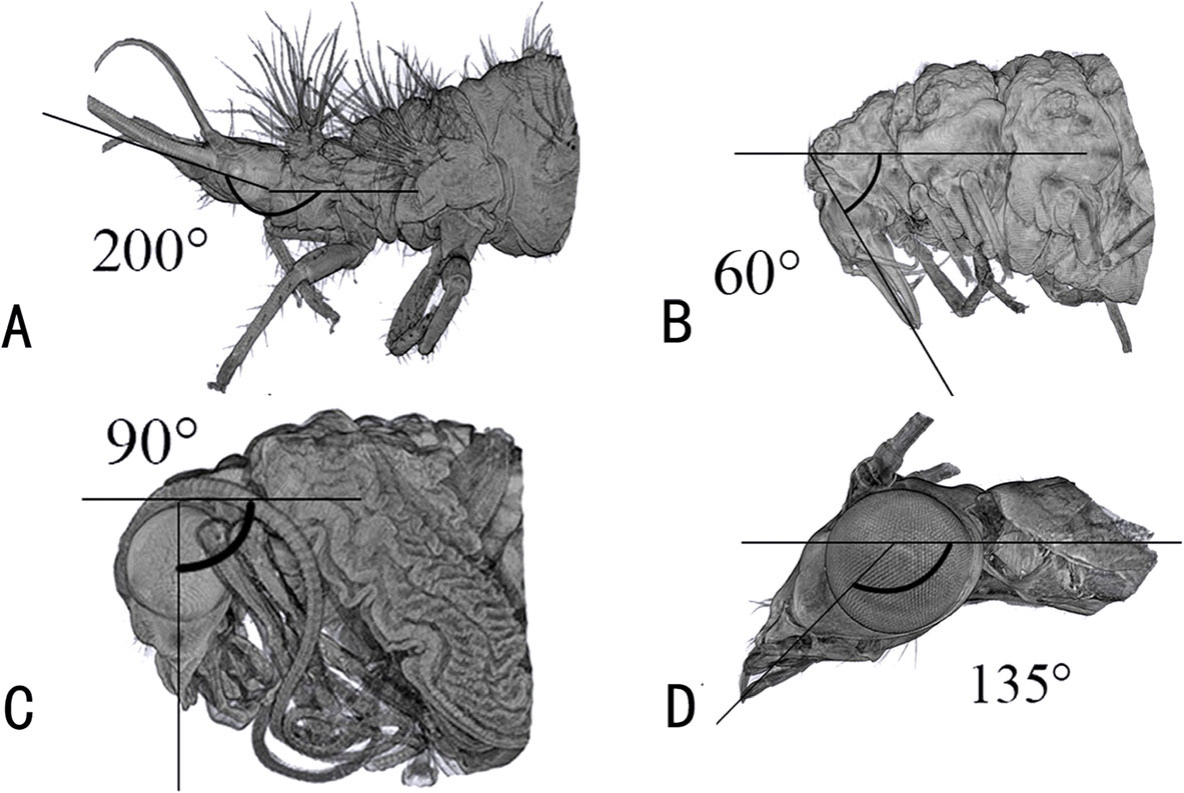
*Chrysopa pallens*, angles between the longitudinal body axis and the longitudinal axis of the mouthparts, 3D reconstructions: **a** larvae, lateral view; **b** prepupae Day 1, lateral view; **c** pupae Day 11, lateral view; **d** adults, lateral view

The pharate adult resembles the adults in almost all skeletal elements except for the absence of the dorsal tentorial arms in the tentorium and the curly elongated antenna. Aside from these skeletal changes, there is one additional major transformation taking place. This is the re-orientation of the head. The larvae are clearly prognathous with an angle of approximately 200° between longitudinal body axis and longitudinal axis of the mouthparts (Fig. 14). The adults are orthognathous with an angle of 135°. The prepupae have the angle 60° from Day 1 to Day 4 (Fig. 2) but the pupae have an angle of 90° from Day 5 to Day 11. The change in the orientation of the mouthparts is fairly sudden and not continuous. The shift from 200° to 60° takes place during cocooning. After the larval cuticle is cracked by Day 5, the angle is 90° and increases once the cocoon starts to break. The angle becomes 135° once the adult emerges. It was suggested by Ge et al. [14] that the “anterior orientation of the mouthparts is a continuous shift rather than a sudden reorientation and takes place more or less continuously during the six days of pupal metamorphosis.” However, Ge et al. only sampled 1 day during pupal development and it is difficult to decide whether the metamorphosis differs in this regard between Neuroptera and Coleoptera.

Based on our scans, we find that the formation of the new cuticle and the histolysis of the internal structures (such as muscles and tentorium) happen during the prepupal stage. After cocooning, the muscle fibers can be recognized easily but they are smaller than in the larvae. Once the new cuticle is formed, and the histolysis of the larval muscles has begun, new muscle granules are presented on Day 4. However, the great increase of the number of granules happens on Day 6. We propose that most muscle bundles would we formed the next day. This is suggested by the data obtained for Day 7 pupae. This order of events is consistent with what is known about honeybees [11] where muscle bundles are present 150 h after the cell is sealed and the muscles begin to break down on Day 3. But our study provides more details on the histolysis of the larval muscles during the first 3 days. We also find that some muscles of the late pupae still appear to be incompletely attached to the skeleton. For example, only one end of the M. craniomandibularis internus (0md1) is attached to sclerites. This phenomenon is also known from Coleoptera [14].

We also find that the modifications of the skeleton happen earlier than the formation of the internal soft parts. This may be due to the fact that the muscles need attachment points. Also, even although the compound eyes are already present in the pharate adults, the optic lobes of the brain are undeveloped until the last day. Based on our study, it will only be fully developed 1 day after emergence. In addition, our study shows that the brain and the suboesophageal ganglion do not disintegrate during the prepupal stage. This indirectly verifies the importance of the central nervous system for the development and metamorphosis. Overall, musculature formation lags behind the changes in the nervous system. We thus conclude that in *Chrysopa*, the modifications of the skeleton come first, is then followed by changes in the nervous system, before the musculature is formed last. All these systems develop gradually during the pupal stage and are only well-developed after emergence. This is consistent with the findings of Oertel [11] in honeybees, a study of leaf beetle metamorphosis [16], and observations on Mecoptera [9]. Based on the phylogenetic relationships between these taxa, we can conclude that this order of events is an ancestral feature of the last common ancestor of Holometabola.

Our findings also suggest that the general belief that the exoskeleton is fully formed at the time of the last larval moult is not correct for green lacewings. The pupa undergoes further changes during the pupal phase. This is consistent with the conclusions of Saltin et al. [9] for Mecoptera, but additional information on other holometabolous orders would be needed in order to test the generality of these processes. Additional species belonging to all major holometabolan clades should be studied. The studies should include all life history stages and use a combination of methods (see [9]).

## Conclusions

Our study shows that muscles are much more slender in larvae than adults which have much more extensive cephalic musculature. Similarly, significant differences are observed for the larval and adult nervous system. The larger brain of adults is presumably needed for processing the signals obtained from the improved sensory organs of adult lacewings which have to reproduce and disperse by flight. For the prepupae and pupae, we demonstrate that neither the inner nor the outer morphology are fully formed at the time of the final larval moult. Instead the morphology of the adult cuticle and the internal structures only form gradually after this moult. The re-modelling of the skeleton finishes first before new muscles are formed. Histolysis and the rebuilding of the skeleton starts with the cephalic organ systems and the central nervous system modifies before the muscle system. Holometabolous insects are morphologically and biologically diverse and this diversity requires a complex developmental system that is flexible enough to produce a wide variety of phenotypes. Unfortunately, the metamorphosis of only very few species has been studied in sufficient detail because it requires the integration of information from morphology, neurobiology, and developmental biology [41–46]. More detailed comparative studies for species representing all major endopterygote clades are needed and the development should be studied with broad range of methods. Such an approach will be needed in order to fully appreciate the remarkable ability of holometaboly to generate numerous phenotypes that is likely to have played a major role in insect diversification [3].

## Methods

### Examined specimens

Although the term “metamorphosis” refers to all transformations during the entire post-embryonic development, we were not able to include eggs, first instar lavae, and second instar lavae in our study due to the low resolution by micro μ-CT. We thus concentrated on the later stages (3rd instar onwards). The last larval instar is the 3rd and was obtained from laboratory culture that was kept at a constant temperature of 25 °C, 16 h of light and 8 h of darkness, and a humidity of around 75%. They were collected in May 2017 from Langfang, Hebei, People’s Republic of China. The larvae and adults were fed with aphids. For this study, the available stages of *Chrysopa pallens* were the last larval stage (stage 3), pupal stage and adults. Three specimens were collected every day from the first prepupal day to the emergence. The adult specimens were killed of 1 day after hatching. Forty specimens were sampled for this study, including 2 larvae, 12 prepupae, 24 pupae, and 2 adults. All materials were preserved in 75% ethanol for less than 24 h before dehydration.

### X-ray computer tomography

All materials used for X-ray micro-computed tomography (μ-CT) were dehydrated in pure n-propanol, then in ethanol solutions from 75 to 100%, stepwise. They were critical-point dried (Leica EM CPD 300). The specimens were scanned by an X-radia Micro CT-520 scanner at Nanjing Institute of Geology and Palaeontology, Chinese Academy of Sciences (beam strength: 40KV, absorption contrast) and X-radia Micro CT-400 scanner at the Institute of Zoology, Chinese Academy of Sciences (beam strength: 60KV, absorption contrast). The resolution of prepupa and pupa is 3.9 ~ 5.8 μm, and that of larva and adult are 2.0 μm.

### Three-dimensional reconstruction (3D)

The muscles and the brains of the larvae, pupae and adults were reconstructed and smoothed with Amira 5.4 based on the obtained image stacks from micro-CT. Final figures were prepared with Adobe Photoshop (CS6).

### Photography

Habit photos of the larvae, pupae and adults were taken by a 5D mark III Canon camera connected to a ZEISS Stemi 2000-c stereoscope (Carl Zeiss, Jena, Germany). The photos are produced using Helicon Focus (ver.6.0.18). Final figures were prepared by Adobe Photoshop (CS6).

### Terminology

The terms used for the head muscles followed the terminology of Wipfler et al. [36].

## Data Availability

All data generated or analyzed during this study are included in this published article.

## Abbreviations

tb: Tentorial bridge
pta: posterior tentorial arms
ata: Anterior tentorial arms
dta: Dorsal tentorial arms
lt: Laminatentorium
sog: suboesophageal ganglion
fg: frontal ganglion

## References

[1] Haug JT (2020). Why the term “larva” is ambiguous, or what makes a larva?. Acta Zool.

[2] Grimaldi D, Engel MS (2005). Insects take to the skies. Evolution of the insects.

[3] Haug JT, Haug C (2013). An unusual fossil larva, the ontogeny of achelatan lobsters, and the evolution of metamorphosis. Bull Geosci.

[4] Barnes RSK, Calow P, Olive PJW (1993). The invertebrates – a new synthesis.

[5] Truman JW, Riddiford LM (1999). The origins of insect metamorphosis. Nature..

[6] Tan J, Hua B (2008). Morphology of immature stages of *Bittacus choui* (Mecoptera: Bittacidae) with notes on its biology. J Nat Hist.

[7] Cai L, Hua B (2009). Morphology of the immature stages of *Panorpa qinlingensis* (Mecoptera: Panorpidae) with notes on its biology. Entomologica Fennica.

[8] Beutel RG, Friedrich F, Ge S-Q, Yang X-K (2014). Insect morphology and phylogeny: a textbook for students of entomology.

[9] Saltin BD, Haug C, Haug JT (2016). How metamorphic is holometabolous development? Using microscopical methods to look inside the scorpionfly (*Panorpa*) pupa. Spixiana..

[10] Misof B, Liu S, Meusemann K, Peters RS, Donath A, Mayer C (2014). Phylogenomics resolves the timing and pattern of insect evolution. Science..

[11] Oertel E (1930). Metamorphosis in the honeybee. J Morphol.

[12] Breidbach O (1987). The fate of persisting thoracic neurons during metamorphosis of the meal beetle *Tenebrio molitor* (Insecta: Coleoptera). Roux’s Archives of Dev Biol.

[13] Breidbach O (1988). Die Verpuppung des Gehirns. Modell Käferhirn.

[14] Ge S-Q, Hua Y, Ren J, Ślipiński A, Heming B, Beutel RG (2015). Transformation of head structures during the metamorphosis of *Chrysomela populi* (Coleoptera: Chrysomelidae). Arthropod Syst Phylogeny.

[15] Polilov AA, Beutel RG (2009). Miniaturisation effects in larvae and adults of *Mikado* sp. (Coleoptera: Ptiliidae), one of the smallest free-living insects. Arthropod Struct Dev.

[16] Polilov AA, Beutel RG (2010). Developmental stages of the hooded beetle *Sericoderus lateralis* (Coleoptera: Corylophidae) with comments on the phylogenetic position and effects of miniaturization. Arthropod Struct Dev.

[17] Bainbridge SP, Bownes M (1981). Staging the metamorphosis of *Drosophila melanogaster*. J Embryol Exp Morpholog.

[18] Crossley AC (1965). Transformations in the abdominal muscles of the blue blow-fly, *Calliphora erythrocephala* (Meig), during metamorphosis. J Embryol Exp Morpholog.

[19] Lowe T, Garwood RJ, Simonsen TJ, Bradley RS, Withers PJ (2013). Metamorphosis revealed: time-lapse three- dimensional imaging inside a living chrysalis. J R Soc Interface.

[20] Deans AR, Miko I, Wipfler B, Friedrich F (2012). Evolutionary phenomics and the emerging enlightenment of arthropod systematics. Invertebr Syst.

[21] Friedrich F, Matsumura Y, Pohl H, Bai M, Hörnschemeyer T, Beutel RG (2014). Insect morphology in the age of phylogenomics: innovative techniques and its future role in systematics. Entomol Sci.

[22] Wipfler B, Pohl H, Yavorskaya MI, Beutel RG (2016). A review of methods for analysing insect structures – the role of morphology in the age of phylogenomics. Curr Opin Insect Sci.

[23] Brooks SJ, Barnard PC (1990). The green lacewings of the world: a generic review (Neuroptera: Chrysopide). Bull Br Museum (Natural History) Entomol.

[24] Brooks SJ (1997). An overview of the current status of Chrysopidae (Neuroptera) systematics. Deutsche Entomologische Zeitschrift.

[25] Brauer F (1852). Versuch einer Gruppirung der Gattungen in der Zunft Planipennia mit besonderer Rücksicht auf die früheren Stände. Stettiner Entomologische Zeitung.

[26] Hennig W (1981). Insect phylogeny.

[27] New TR (1989). Planipennia. Lacewings. Handbuch der Zoologie (Berlin).

[28] Canard M, Séméria Y, New TR (1984). Biology of Chrysopidae.

[29] Tauber MJ, Tauber CA, Daane KM, Hagen KS (2000). Commercialization of predators: recent lessons from green lacewings (Neuroptera: Chrysopidae: *Chrysoperla*). Am Entomol (and Botanist).

[30] Tauber MJ, Tauber CA, Albuquerque GS, Resh VH, Cardé R (2009). Neuroptera (lacewings, antlions). Encyclopedia of insects.

[31] McEwen P, New TR, Whittington AE (2001). Lacewings in the crop environment.

[32] Tauber CA (2014). Apochrysinae (Neuroptera: Chrysopidae): New larval description and subfamilial comparisons. Zootaxa.

[33] Tauber CA, Sosa F, Albuquerque GS, Tauber MJ (2013). Adults and larvae of two *Leucochrysa* (*Leucochrysa*) species (Neuroptera: Chrysopidae): descriptions, biological notes, and relationships. Zootaxa..

[34] Tauber CA, Winterton SL (2014). Third instar of the myrmecophilous *Italochrysa insignis* (Walker) from Australia (Neuroptera: Chrysopidae: Belonopterygini). Zootaxa..

[35] Miller FW (1933). Musculature of the lacewing (*Chrysopa florabunda*) (Neuroptera). J Morphol.

[36] Wipfler B, Machida R, Mueller B, Beutel RG (2011). On the head morphology of Grylloblattodea (Insecta) and the systematic position of the order, with a new nomenclature for the head muscles of *Dicondylia*. Syst Entomol.

[37] Beutel RG, Friedrich F, Hörnschemeyer T, Pohl H, Hünefeld F, Beckmann F (2011). Morphological and molecular evidence converge upon a robust phylogeny of the megadiverse Holometabola. Cladistics..

[38] von Kéler S (1963). Entomologisches Wörterbuch.

[39] Winterton SL, Hardy NB, Wiegmann BM (2010). On wings of lace: phylogeny and Bayesian divergence time estimates of Neuropterida (Insecta) based on morphological and molecular data. Syst Entomol.

[40] Beutel RG, Friedrich F, Aspöck U (2010). The larval head of Nevrorthidae and the phylogeny of Neuroptera (Insecta). Zool J Linnean Soc.

[41] Hartenstein V (1993). Atlas of drosophila development. In: bate M, arias AM, editors. The development of *Drosophila melanogaster*.

[42] Heming BS (2003). Insect development and evolution. Comstock publishing associates.

[43] Sehnal F (1985). Morphology of insect development. Annu Rev Entomol.

[44] Nüsch H (1987). Metamorphose bei Insekten: direkte und indirekte Entwicklung bei Apterygoten and Exopterygoten. Zool Jahrb Abt Anat Ontog Tiere.

[45] Svácha P (1992). What are and what are not imaginal discs: re-evaluation of some basic concepts (Insecta, Holometablola). Dev Biol.

[46] Sehnal F, Svácha P, Zrzavy J, Gilbert LI, Tata JR, Atkinson BG (1996). Evolution of insect metamorphosis. Metamorphosis. Postembryonic reprogramming of gene expression in amphibian and insect cells.

